# Latin American consumption of major food groups: Results from the ELANS study

**DOI:** 10.1371/journal.pone.0225101

**Published:** 2019-12-26

**Authors:** Irina Kovalskys, Attilio Rigotti, Berthold Koletzko, Mauro Fisberg, Georgina Gómez, Marianella Herrera-Cuenca, Lilia Yadira Cortés Sanabria, Martha Cecilia Yépez García, Rossina G. Pareja, Ioná Zalcman Zimberg, Ana Del Arco, Luciana Zonis, Agatha Nogueira Previdelli, Viviana Guajardo, Luis A. Moreno, Regina Fisberg, the ELANS Study Group

**Affiliations:** 1 Nutrition, Health and Wellbeing Area, International Life Science Institute (ILSI-Argentina), Buenos Aires, Argentina; 2 Pontifica Universidad Catolica Argentina Facultad de Medicina, Buenos Aires, Argentina; 3 Departamento de Nutrición, Diabetes y Metabolismo, Centro de Nutrición Molecular y Enfermedades Crónicas, Escuela de Medicina, Pontificia Universidad Católica, Santiago, Chile; 4 Ludwig-Maximilians-Universität Munich, Division of Metabolic and Nutritional Medicine, Dr. von Hauner Children's Hospital, University of Munich Medical Center, Munich, Germany; 5 Instituto Pensi, Fundação Jose Luiz Egydio Setubal, Hospital Infantil Sabara, São Paulo, Brazil; 6 Departamento de Pediatria, Universidade Federal de São Paulo, São Paulo, Brazil; 7 Departamento de Bioquímica, Escuela de Medicina, Universidad de Costa Rica, San José, Costa Rica; 8 Centro de Estudios del Desarrollo, Universidad Central de Venezuela (CENDES-UCV)/Fundación Bengoa, Caracas, Venezuela; 9 Departamento de Nutrición y Bioquímica, Pontificia Universidad Javeriana, Bogotá, Colombia; 10 Colegio de Ciencias de la Salud, Universidad San Francisco de Quito, Quito, Ecuador; 11 Instituto de Investigación Nutricional, Lima, Peru; 12 Departamento de Psicobiologia, Universidade Federal de São Paulo, São Paulo, Brazil; 13 Faculdade de Ciências Biológicas e da Saúde, Universidade São Judas Tadeu, São Paulo, Brazil; 14 Instituto de Investigación Sanitaria Aragón (IIS Aragón), Centro de Investigación Biomédica en Red Fisiopatología de la Obesidad y Nutrición (CIBERObn), University of Zaragoza, Zaragoza, Spain; 15 GENUD (Growth, Exercise, Nutrition and Development) Research Group, Instituto Agroalimentario de Aragón (IA2), University of Zaragoza, Zaragoza, Spain; 16 Departmento de Nutrição, Faculdade de Saúde Pública, Universidade de São Paulo, São Paulo, Brazil; BITS Pilani, INDIA

## Abstract

**Background:**

The Latin American (LA) region is still facing an ongoing epidemiological transition and shows a complex public health scenario regarding non-communicable diseases (NCDs). A healthy diet and consumption of specific food groups may decrease the risk of NCDs, however there is a lack of dietary intake data in LA countries.

**Objective:**

Provide updated data on the dietary intake of key science-based selected food groups related to NCDs risk in LA countries.

**Design:**

ELANS (Latin American Study of Nutrition and Health) is a multicenter cross-sectional study assessing food consumption from an urban sample between15 to 65 years old from 8 LA countries (Argentina, Brazil, Chile, Colombia, Costa Rica, Ecuador, Peru, and Venezuela). Two 24-HR were obtained from 9,218 individuals. The daily intake of 10 food groups related to NCDs risk (fruits; vegetables; legumes/beans; nuts and seeds; whole grains products; fish and seafood; yogurt; red meat; processed meats; sugar-sweetened beverages (ready-to-drink and homemade)) were assessed and compared to global recommendations.

**Results:**

Only 7.2% of the overall sample reached WHO’s recommendation for fruits and vegetables consumption (400 grams per day). Regarding the dietary patterns related to a reduced risk of NCDs, among the overall sample legumes and fruits were the food groups with closer intake to the recommendation, although much lower than expected (13.1% and 11.5%, respectively). Less than 3.5% of the sample met the optimal consumption level of vegetables, nuts, whole grains, fish and yogurt. Largest country-dependent differences in average daily consumption were found for legumes, nuts, fish, and yogurt. Mean consumption of SSB showed large differences between countries.

**Conclusion:**

Diet intake quality is deficient for nutrient-dense food groups, suggesting a higher risk for NCDs in the urban LA region in upcoming decades. These data provide relevant and up-to-date information to take urgent public health actions to improve consumption of critically foods in order to prevent NCDs.

## Introduction

The Latin American region shows one of the most complex public health scenarios of the world regarding the increasing incidence of non-communicable diseases (NCDs) [1]. Thus, urgent calls from the World Health Organization (WHO) and the Pan American Health Organization (PAHO) are focused on taking actions in Latin American countries to attenuate all preventable risk factors, as well as morbidity and mortality due to obesity, type 2 diabetes, cardiovascular diseases, and cancer [2].

One of the major scientific concerns is the role of diet and the potential association of some specific dietary determinants with health and diseases [3]. Beyond specific nutrients, it is important to identify foods and food groups that may promote health or increase the risk of diet-related diseases. Food and food groups-based recommendations are the optimal approach for dietary guidelines, being increasingly based on food patterns, rather than nutrient composition [4]. High intake of fruits, vegetables, fish (including seafood), whole grains and nuts in association with low consumption of sugar-sweetened beverages (SSB) and red and processed meat has been associated with decreased risk for NCDs, regardless of the country, ethnic background or culture [4–23].

Despite the widely agreed importance of having updated dietary intake data worldwide, in Latin American countries there is still a lack of information. During recent years, global nutritional information has been generated by the application of robust statistical methods and models based on available data [7, 21, 23]. The Global Burden of Disease Study (GBD) [21, 23] has evaluated the main risk factors, including dietary determinants, associated with deaths and disability-adjusted life years since 1990 [21, 23]. Using national surveys and national food balance sheets, the Nutrition and Chronic Diseases Expert Group (NutriCoDE), as part of GBD, built a Global Dietary Database of Consumption to evaluate global data on food intake based on information systematically collected using equivalent and standardized methods. Their method included de-identified national data sets with the goal of generating "comparable estimates of consumption of food around the world" [7]. In addition, NutriCoDE identified the optimal consumption of 10 food groups (fruits, vegetables, legumes/beans, nuts/seeds, whole grains, fish/seafood, yogurt, unprocessed red meats, processed meats and sugar-sweetened beverages) associated with beneficial cardio-metabolic effects and reduced chronic diseases risk [8].

Despite these efforts, the Latin American population is usually misrepresented in these global food intake studies as a consequence of limited available datasets. In addition, NutriCoDE described Latin America as one of the regions with greater statistical uncertainty due to the low quality of the available data [7]. Furthermore, Latin American countries are facing limited national budgets, other health priorities (e.g., vaccination, infectious diseases, reproductive health, etc.) resulting in limited epidemiological surveys on environmental risk factors for NCDs. Indeed, Latin America is still facing an ongoing epidemiological transition that leads to the coexistence of two opposite nutrition-related issues: undernutrition together with overweight/obesity [24–30]. Thus, updating information on NCDs and their risk conditions remains critical for taking evidence-based public health actions at the individual and community levels within the countries of this region.

The *Estudio Latinoamericano de Nutrición y Salud* (ELANS, Latin American Study of Nutrition and Health) provides an opportunity to fill some of these gaps. ELANS is a multicenter cross-sectional nutrition, physical activity, and health survey with a nationally representative sample of urban populations from eight Latin American countries (Argentina, Brazil, Chile, Colombia, Costa Rica, Ecuador, Peru, and Venezuela), developed using a rigorous protocol of standardization to harmonize a shared food composition database generating comparable dietary intake data among Latin American countries with minimum random and systematic errors [31, 32].

Thus, the aim of this study was to provide updated data on the dietary intake of key food groups reported to be related to NCDs risk in Latin American countries, verify possible differences according to country, age, gender and sociodemographic factors, and to compare the intake of these major food groups to the current recommendations.

## Material and methods

### Study sample

**Fig 1:**
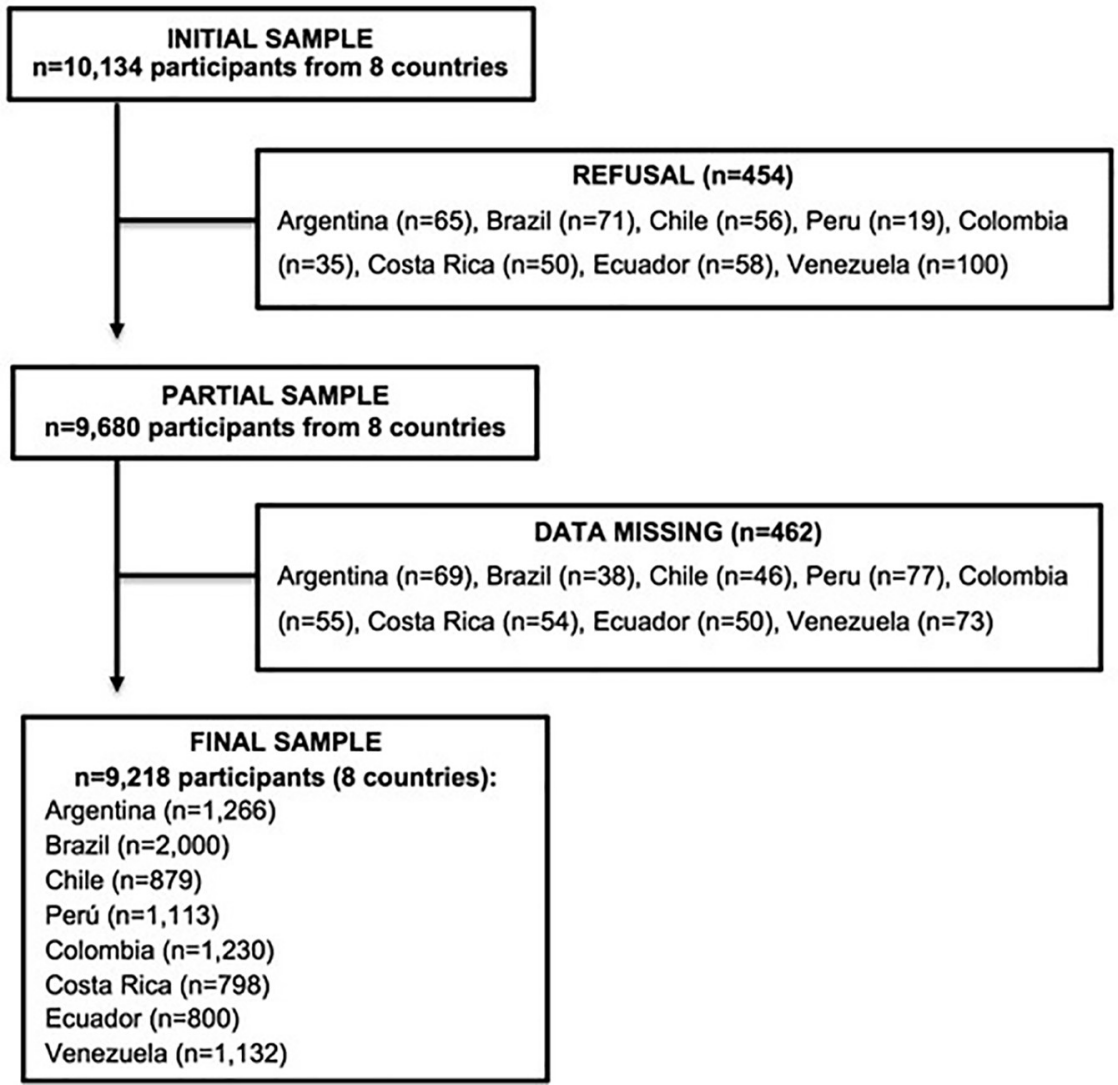
Flow chart of study population. ELANS sample of the urban population.

ELANS is a multicenter cross-sectional study that evaluated simultaneously household-based individual food consumption, physical activity and sociodemographic characteristics among eight Latin American countries, using a standardized methodology. Data were collected from September 2014 to August 2015. Available sociodemographic information was considered to obtain nationally representative samples of the urban populations from these Latin American countries, where 80–90% of the population is living. The total sample was composed of 9,218 subjects from 15 to 65 years old (Argentina, Brazil, Chile, Colombia, Costa Rica, Ecuador, Peru, and Venezuela) stratified by age, gender, socioeconomic level and geographical location (Fig 1). It was a random complex multistage sample, stratified by geographical region, sex, age and socioeconomic level (SEL), with a random selection of Primary Sampling Units (PSU) and Secondary Sampling Units (SSU). For the selection of households within each SSU, households were selected through systematic randomization. Selection of the respondent within a household was done using 50% of the sample next birthday, 50% last birthday, controlling quotas for gender, age, and SEL. This complex multistage sample was established with a confidence level of 95% and a maximum error of 3.49%. Sample weighting was applied at each country level accounting for key variables of interest (the geographical region, sex, age and SEL). No sample weighting was applied after the eight countries’ database was unified due to the lack of official data of the urban population distribution in Latin America. This single database was used for all analysis presented in the current paper.

The ELANS protocol was approved by the Western Institutional Review Board (#20140605) and registered at Clinical Trials (#NCT02226627). It was also approved by a regional Ethics committee in each country. All participants gave their informed consent/assent before participation in the survey. More detailed information on this study design, protocol and methodology was previously published [31].

### Dietary intake

The standard study protocol to investigate dietary intake in all countries has been previously reported [31, 32]. Briefly, the protocol included two 24-hour recalls (24-HR) using the Multiple Pass Method [33] applied by trained interviewers face-to-face during two household visits on non-consecutive days, with an interval of up to 8 days between them. The 24-h recalls included both weekdays and weekend days, with a proportional distribution of days among the sample, in order to capture the day-to-day variation intake. The households 24-h recalls were supervised by trained nutritionists who were also responsible for converting the measures obtained into grams and milliliters. Dietary data collection was thoroughly harmonized in each country, considering the nutritional equivalency of local and traditional foods items to USDA composition table foods available through the Nutrition Data System for Research program (NDS-R, version 2013) database. Thus, a food matching standardized procedure was conducted by professional nutritionists in each country in order to minimize errors and verify quantities of key nutrients, the complete procedure for standardization of the food composition database which has been described in detail elsewhere [32]. Many regional and commercial food and beverages not available in NDS-R database were broken down into ingredients and entered into the software as user recipes. All food and beverages were computed in grams/day.

The two 24-h recalls were used to estimate usual food consumption and to evaluate intra-individual variability in nutrient intake. The web-based statistical modeling technique Multiple Source Method (MSM) (https://msm.dife.de/), proposed by the European Prospective Investigation into Cancer and Nutrition (EPIC), was used to estimate energy and nutrients intake [34]. To minimize errors derived from the method, the estimation of usual intake was conducted individually for each country, thus taking into account differences in eating habits among the Latin American populations. In the present study, the dietary intake was analyzed in terms of food groups, not nutrients. The daily intake of each food group for each participant was estimated considering the mean consumption from both records.

### Food grouping

A total of 2,278 different types of food and beverages were reported in both 24-HR. They were grouped into ten categories as detailed shown in Table 1: fruits; vegetables; legumes/beans; nuts and seeds; whole grains products; fish and seafood; yogurt; red meat; processed meats; sugar-sweetened beverages (ready-to-drink and homemade) (See S1 Text).

**Table 1:** Composition of the ten food groups related to NCDs.

Food Groups	Foods and beverages included
**Fruits (excluded fruit juice)**	All kinds of fruits, excluding the ones used to prepare natural juice (e.g.: mango, papaya, apples, pear, banana)
**Vegetables**	Vegetables and green leaves (e.g.: tomato, kale, cucumber, okra, arugula, cauliflower, broccoli, squash)
**Legumes/Beans**	Beans, chicken peas, green peas
**Nuts and Seeds**	Nuts (e.g.: almonds, Brazil nuts, cashew, peanuts, pecans, walnuts) and Seeds (e.g.: pumpkin seeds, sunflower, flaxseeds, sesame seed, linseed, quinoa)
**Whole Grains products**	Cookies, crackers, bread, whole pasta, brown rice, whole flour, breakfast cereal, oats
**Fish and Seafood**	Fish (e.g.: tuna, white fish, salmon, canned fish (sardine)) and Seafood (e.g.: clams, mussel, crab, snails, oyster, lobster, prawns, shrimp)
**Yogurt**	High and low-fat yogurt
**Red Meat**	Pork meat, beef, lamb
**Processed Meats**	Hamburger, meatballs, sausage, bacon, ham, bologna, salami. Included cold cuts.
**Sugar-Sweetened Beverages (SSB):**	
**SSB purchased ready-to-drink**	All kind of ready-to-drink beverage with added sugar (e.g.: powder juice, nectar, sodas, energy drinks, teas, flavored water)
**SSB homemade (or do not purchased ready-to-drink)**	All kind of homemade beverage with added sugar (e.g.: tea, coffee, milk, mate, fruit juices)

The food/beverage group definitions were primarily based on the NDS-R software food groups, which were based on the 1992 U.S. Department of Agriculture Food Guide Pyramid [35]. Briefly, NDS-R codes foods using the grouping system developed at Tufts University for application in the USDA database and NDS-R system. In its most extensive format, there are 457 different groups designed for flexibility in its use, including easy regrouping for specific purposes (e.g., low fat vs. full fat versions, specific groups of vegetables, etc.). These groups were condensed into a shorter list of ten food/beverage groups, in which foods and beverages were combined according to nutritional similarities as well as their positive or negative effects on health or diseases, mainly on cardiovascular diseases (CVD) and other mortality causes based on scientific evidence provided by several meta-analyses [4–6, 8–20, 22, 36–45].

The optimal consumption level of the major food groups was based on 1. WHO’s recommendations [46] (global reference) for fruits and vegetables and 2. The minimum daily requirement evidenced by the literature (three meta-analyses and systematic reviews on food groups consumption and NCDs [4, 5, 8]) to decrease the relative risk for CVD and/or mortality (defined in the "range of consumption required"). The chosen values for a range of optimal consumption of the different food groups related to NCDs risk are shown in Table 2.

**Table 2:** Range of optimal consumption adopted for the major food groups.

Food Groups	Quantity (grams or milliliters) observed in the scientific literature—Cut-offs	Range of "optimal consumption" adopted
*Decrease Relative Risk*		
**Fruits**	400 g/day (+vegetables)^a^; 300 g/day^b^; 250–300 g/day (10%)^c^; 200 g/day (15–20%)^d^	200–400 g/day
**Vegetables**	400 g/day (+fruits)^a^; 400 g/day (+legumes/beans)^b^; 300 g/day (11%)^c^; 400 g/day (12%)^d^	300–400 g/day
**Legumes/Beans**	100 g/day^b^; 150 g/day (16%)^c^; 100 g/day (10%)^d^	100–150 g/day
**Nuts and Seeds**	141.75 g/week^b^; 15–20 g/day (17%)^c^; 10–15 g/day (21%)^d^	10–20 g/day
**Whole Grains**^a^	125 g/day^b^; 100 g/day (25%)^c^; 100 g/day (17%)^d^	100–125 g/day
**Fish and Seafood**	350 g/week^b^; 200 g/day (10%)^c^; 250 g/day (15%)^d^	200–250 g/day
**Yogurt**	610 g/week^b^; 200 g/day^e^	200 g/day
*Dose-dependent effect on Relative Risk*		
**Red meat**	100g/week^b^; 0.5 serving/day^f^; 100g/day (increase RR = 10–20%)^d^	50–100 g/day
*Increase Relative Risk*		
**Processed meats**	0^b^; 200 g/day (60%)^c^; 70 g/day (15–25%)^d^	N/A
**SSB**	0^b^; 250 mL/day (7%)^c^; 500 mL/day (16–35%)^d^	N/A

Data are according to global reference and/or evidence related to NCDs. N/A: not applicable

^a^ WHO Guidelines [46].

^b^ Adapted from Micha et al [8].

^c^ Adapted from Schwingshackl et al [4].

^d^ Adapted from Bechthold et al [5].

^e^ Adapted from Wu et al [10].

^f^ Adapted from O'Connor et al [14].

Description and evidence of chosen food groups are described in detail in the S1 Text.

### Statistical analysis

Analyses were based on the consumption of grams of foods since the current recommendations are based on grams of food/food groups per day. As all participants provided two 24-HR, the daily intake of each food group was estimated considering the mean consumption from both records. Analysis of food groups consumption was made by central tendency (mean and standard deviation (SD)) and distribution statistics (percentiles) analyzed by country, gender, age group, and SEL.

The prevalence of adequate intake based on global and WHO recommendations was calculated as the percentage of individuals with mean daily intake equal to or higher than the current recommendation for each food group.

Additional analysis to identify the daily portion size of each food group was made for those individuals who reported positive intake of these food groups in one or both 24-HR (identified as ‘consumers’). Therefore, those food groups which were not consumed by the participant (in one or both records) were not included in the analysis. In other words, intake equal “zero” was considered as a missing value. Excluding the non-consumers avoided underestimating the real portion size.

The Kruskal-Wallis test was used to compare the consumption of each key food group (in grams) across the countries, considering both population- and individual-level intake. This test was also performed to identify statistical differences between consumption and SEL (low, middle and high) within each countryby using the Kruskal-Wallis test.

All analyses were performed using SPSS software (version 22.0; SPSS Inc, Chicago, IL, USA). A p-value lower than 0.05 was considered as statistically significant.

## Results

**Fig 2:**
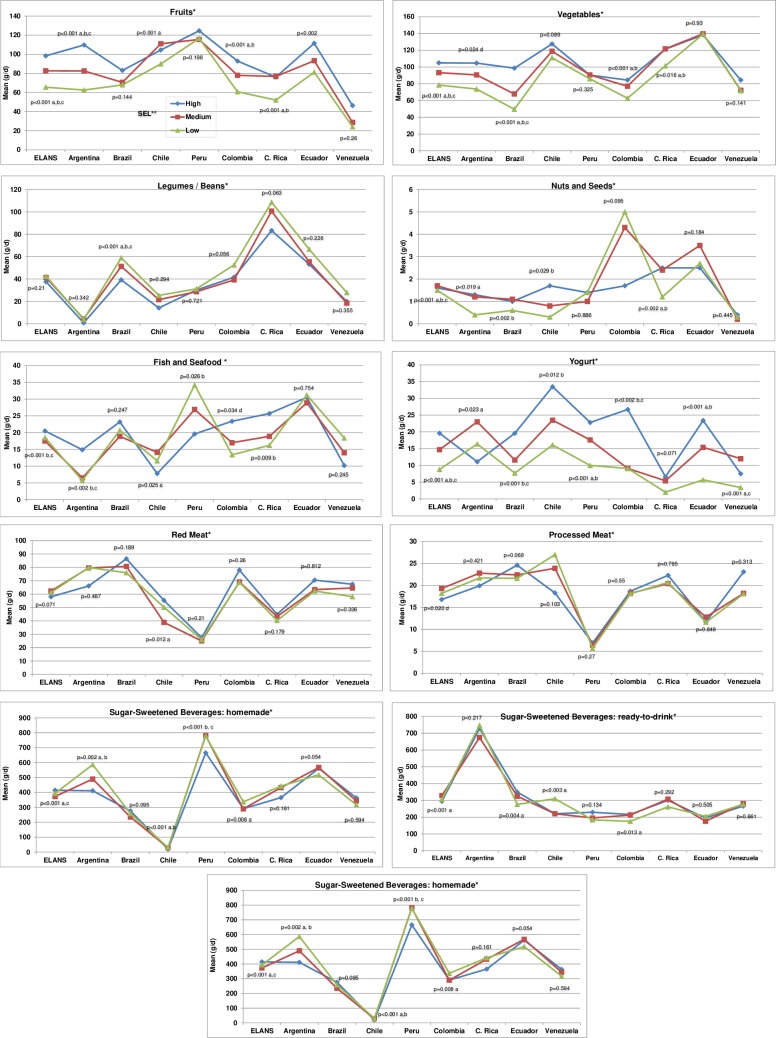
Overall consumption of the major food groups by country and socioeconomic level. *Food Groups: food or beverages with demonstrated associations with NCDs. Nationally representative data from the urban population, 15–65 years old, obtained from the ELANS by the average of two non-consecutive 24-HR, ** SEL: socioeconomic level: H: High; M: Middle; L: Low. p values by Kruskal Wallis test: a low vs middle (p<0.05); b low vs high (p<0.05); c middle vs high (p<0.05)–all adjusted for multiple testing (using the Bonferroni error correction). d No statistical differences were found after comparisons between groups, adjusted for multiple testing.

Mean intake (grams per day ± SD) of the major food groups stratified by country, gender and age group is shown in Table 3 and by country and SEL in Fig 2. Mean consumption of fruits in total ELANS population was 75.3 g/day (26.2 g/day in Venezuela—117.8 g/day in Peru). Fruit consumption tended to be higher for women, and increase with age and SEL in most countries. Remarkably, older adults’ intake was higher than that of those who were adolescents (95 g/day vs 64.4 g/day) and fruit intake in many countries was significantly associated with high SEL.

**Table 3:** Comparison of dietary intake of the ten major food groups in individuals residing in urban areas of Latin America, according to the country of living, gender and age.

Food Groups*	Country	N	Mean g/day (SD)		p**	Gender Mean g/day (SD)		Age group Mean g/day (SD)			
						Male	Female	15–19.9	20–34.9	35–49.9	50–65.9
**Fruits**	**ELANS**	9218	75.3	(25.2)	<0.001	71.9 (123.3)	78.4 (126.8)	64.4 (123.6)	67.7 (117.0)	75.8 (129.9)	95.7 (131,6)
	**Argentina**	1266	74.3	(111.5)		70.0 (116.9)	77.8 (106.7)	63.6 (111.1)	60.5 (99.5)	70.0 (101.3)	106.7 (133.7)
	**Brazil**	2000	70.5	(112.9)		65.8 (111.3)	74.6 (114.1)	48.4 (96.8)	56.8 (103.0)	72.6 (100.6)	104.6 (143.7)
	**Chile**	879	100.5	(122.1)		102.4 (127.5)	98.8 (116.9)	101.9 (116.1)	91.9 (106.1)	95.9 (122.8)	118.6 (144.2)
	**Peru**	1113	117.8	(155.3)		116.0 (151.5)	119 (158.7)	103.3 (192.2)	123.5 (156.4)	111.8 (141.4)	125.8 (136.4)
	**Colombia**	1230	67.9	(120.6)		63.6 (127.6)	72.1 (113.3)	62.8 (93.3)	65.2 (136.3)	71.1 (118.7)	71.0 (109.7)
	**Costa Rica**	798	68.6	(115.5)		65.8 (123.0)	71.3 (107.8)	48.9 (102.0)	63.6 (119.8)	72.2 (113.5)	88.8 (117.7)
	**Ecuador**	800	89.6	(169.8)		82.9 (131.5)	96.3 (200.6)	81.5 (160.4)	77.4 (111.8)	95.2 (239.9)	116.9 (149.3)
	**Venezuela**	1132	26.2	(73.7)		24.3 (76.9)	27.9 (70.5)	19.2 (45.3)	18.8 (52.2)	32.7 (101.1)	38.2 (80.3)
**Vegetables**	**ELANS**	9218	86.7	(87.3)	<0.001	90.5 (86.2)	83.1 (88.1)	76.4 (80.7)	83.3 (81.4)	90.9 (91.3)	93.5 (94.8)
	**Argentina**	1266	83.1	(87.8)		79.3 (75.5)	86.2 (96.8)	71.3 (71.9)	78.4 (81.6)	85.4 (94.5)	93.5 (94.5)
	**Brazil**	2000	62.1	(96.9)		60.7 (89.7)	63.3 (102.9)	47.7 (90.7)	51.4 (67.3)	70.6 (110.1)	77.0 (119.3)
	**Chile**	879	116.0	(80.6)		114.8 (81.5)	117.2 (79.8)	96.3 (76.0)	115.6 (85.2)	122.7 (82.2)	120.0 (72.1)
	**Peru**	1113	88.3	(57.0)		97.7 (60.0)	80.0 (52.9)	83.1 (56.5)	90.8 (58.9)	88.4 (56.2)	86.5 (54.2)
	**Colombia**	1230	68.3	(71.9)		73.5 (73.5)	63.4 (70.0)	56.8 (65.0)	61.7 (67.0)	73.4 (68.6)	78.2 (83.1)
	**Costa Rica**	798	115	(109.8)		117.5 (99.9)	112.5 (118.8)	96.0 (88.9)	115.2 (120.3)	116.8 (100.0)	126.9 (115.8)
	**Ecuador**	800	139.4	(90.1)		162.1 (97.7)	116.9 (75.6)	130.5 (94.5)	139.4 (82.7)	147.5 (99.9)	134.2 (84.9)
	**Venezuela**	1132	72.4	(62.4)		75.4 (66.6)	69.5 (58.1)	61.0 (60.8)	72.5 (61.7)	74.0 (55.9)	78.2 (73.2)
**Legumes/ Beans**	**ELANS**	9218	41.6	(65.3)	<0.001	49.0 (74.2)	34.8 (55.1)	42.3 (64.6)	41.3 (62.1)	43.6 (72.7)	38.7 (60.5)
	**Argentina**	1266	4.0	(19.4)		3.2 (17.9)	4.6 (20.5)	1.9 (9.3)	3.6 (20.0)	4.3 (18.5)	5.2 (23.0)
	**Brazil**	2000	53.8	(48.4)		67.7 (54.4)	41.3 (38.2)	58.9 (48.8)	53.7 (47.6)	55.3 (52.4)	48.8 (42.7)
	**Chile**	879	22.6	(47.3)		24.4 (51.5)	20.9 (42.9)	14.9 (30.3)	23.6 (53.5)	21.2 (45.4)	27.4 (47.3)
	**Peru**	1113	30.1	(44.6)		34.9 (51.5)	25.9 (37.0)	33.5 (53.3)	28.9 (40.6)	31.5 (45.9)	28.1 (43.7)
	**Colombia**	1230	47.8	(78.3)		50.1 (81.8)	45.7 (74.8)	44.4 (71.8)	46.5 (74.3)	53.8 (91.2)	45.0 (71.2)
	**Costa Rica**	798	100.9	(95.0)		131.0 (107.6)	71.6 (69.5)	101.5 (90.3)	103.7 (86.3)	104.0 (110.6)	90.5 (90.5)
	**Ecuador**	800	60.7	(79.6)		64.3 (84.9)	57.1 (74.0)	56.8 (79.9)	63.7 (75.6)	59.0 (84.1)	59.8 (81.6)
	**Venezuela**	1132	25.8	(61.2)		25.9 (66.4)	25.6 (55.9)	27.1 (56.8)	20.8 (46.5)	32.7 (82.0)	25.4 (55.3)
**Nuts and Seeds**	**ELANS**	9218	1.6	(12.9)	<0.001	1.7 (14.0)	1.5 (11.7)	1.2 (11.3)	1.6 (11.3)	1.9 (16.3)	1.5 (11.1)
	**Argentina**	1266	0.8	(6.1)		0.8 (7.6)	0.8 (4.4)	1.2 (12.2)	1.0 (5.5)	0.4 (3.9)	0.7 (4.1)
	**Brazil**	2000	0.9	(6.1)		0.9 (7.3)	0.8 (4.9)	0.2 (1.5)	0.8 (4.7)	0.9 (5.0)	1.6 (10.2)
	**Chile**	879	0.6	(3.5)		0.6 (3.7)	0.7 (3.4)	0.4 (1.9)	1.0 (4.6)	0.3 (2.1)	0.7 (3.8)
	**Peru**	1113	1.3	(14.6)		0.7 (4.7)	1.7 (19.6)	0.6 (3.1)	0.9 (5.0)	2.5 (27.4)	0.9 (4.4)
	**Colombia**	1230	4.6	(24.7)		5.2 (28.0)	3.9 (21.0)	5.0 (28.1)	4.0 (22.9)	6.0 (27.1)	3.6 (22.6)
	**Costa Rica**	798	2.0	(10.7)		2.4 (13.2)	1.7 (7.6)	0.9 (5.9)	2.7 (13.4)	1.8 (10.7)	1.8 (7.3)
	**Ecuador**	800	3.0	(18.8)		3.7 (23.2)	2.2 (13.2)	2.0 (7.8)	3.2 (17.0)	4.2 (27.6)	1.5 (10.2)
	**Venezuela**	1132	0.3	(3.8)		0.1 (1.9)	0.4 (5.0)	0.0 (0.1)	0.4 (5.6)	0.3 (2.5)	0.1 (1.2)
**Whole Grains**	**ELANS**	9218	13.1	(31.3)	<0.001	13.2 (35.9)	12.9 (26.4)	13.2 (31.2)	12.7 (30.5)	12.5 (30.8)	14.4 (33.4)
	**Argentina**	1266	13.5	(32.1)		14.1 (38.8)	13.1 (25.3)	10.6 (34.5)	12.4 (27.2)	13.9 (36.3	16.4 (32.1)
	**Brazil**	2000	13.6	(33.1)		13.9 (38.7)	13.3 (27.3)	16.8 (37.7)	13.6 (35.3)	12.9 (29.9)	12.8 (30.9)
	**Chile**	879	10.6	(25.8)		9.7 (25.8)	11.5 (25.8)	15.8 (28.2)	10.9 (24.4)	8.3 (23.4)	10.1 (28.9)
	**Peru**	1113	14.7	(27.8)		15.8 (33.1)	13.8 (22.0)	19.0 (42.0)	14.0 (27.0)	12.4 (19.9)	16.4 (24.2)
	**Colombia**	1230	14.6	(38.5)		14.7 (43.6)	14.5 (32.9)	10.0 (21.5)	13.9 (34.7)	14.4 (35.5)	18.1 (51.4)
	**Costa Rica**	798	14.9	(28.6)		15.9 (33.4)	14.0 (22.9)	12.1 (23.8)	15.5 (29.6)	14.9 (31.4)	16.3 (25.7)
	**Ecuador**	800	12.2	(36.9)		12.3 (42.9)	12.1 (29.9)	9.5 (26.5)	10.7 (37.0)	14.2 (45.1)	15.0 (29.9)
	**Venezuela**	1132	9.5	(20.8)		8.4 (19.4)	10.5 (22.0)	9.6 (18.6)	10.0 (21.9)	8.6 (18.0)	9.6 (23.8)
**Fish and Seafood**	**ELANS**	9218	18.3	(51.9)	<0.001	19.9 (49.2)	16.9 (54.2)	15.5 (38.3)	18.0 (47.0)	20.0 (65.6)	18.3 (46.2)
	**Argentina**	1266	6.5	(26.9)		7.3 (31.9)	5.9 (21.9)	5.6 (22.7)	4.6 (19.6)	8.6 (35.3)	7.4 (25.6)
	**Brazil**	2000	20.1	(82.1)		19.4 (66.2)	20.7 (94.0)	12.7 (46.6)	19.6 (67.9)	22.2 (115.2)	22.0 (59.5)
	**Chile**	879	12.4	(29.1)		11.2 (28.8)	13.4 (29.3)	12.5 (26.3)	11.7 (29.6)	13.6 (29.0)	11.7 (30.0)
	**Peru**	1113	28.9	(51.4)		31.8 (56.8)	26.3 (46.0)	29.7 (52.3)	28.3 (52.9)	30.4 (47.1)	27.3 (53.9)
	**Colombia**	1230	15.1	(38.2)		17.5 (42.5)	12.7 (33.3)	8.0 (25.6)	14.1 (38.3)	18.4 (38.7)	16.3 (42.1)
	**Costa Rica**	798	19.0	(35.1)		21.2 (35.9)	16.8 (34.2)	14.3 (28.2)	20.0 (34.1)	21.7 (39.7)	16.5 (34.7)
	**Ecuador**	800	30.3	(46.6)		34.5 (48.3)	26.1 (44.5)	26.8 (40.7)	30.0 (47.1)	32.3 (46.3)	30.8 (51.2)
	**Venezuela**	1132	17.2	(39.7)		20.3 (45.3)	14.2 (33.2)	15.5 (36.1)	16.8 (36.8)	16.3 (38.3)	20.9 (49.3)
**Yogurt**	**ELANS**	9218	12.1	(46.5)	<0.001	10.8 (47.2)	13.2 (45.8)	18.2 (62.9)	13.7 (48.9)	8.7 (36.8)	10.0 (40.8)
	**Argentina**	1266	19.2	(67.1)		20.3 (79.8)	18.3 (54.4)	31.3 (114.4)	23.2 (72.8)	13.9 (46.3)	13.5 (41.7)
	**Brazil**	2000	10.5	(44.3)		8.4 (39.2)	12.4 (48.3)	14.0 (46.7)	10.0 (39.5)	9.2 (46.5)	11.3 (47.8)
	**Chile**	879	20.9	(50.5)		20.1 (50.4)	21.6 (50.6)	42.0 (70.6)	26.3 (57.8)	10.4 (32.4)	13.5 (36.7)
	**Peru**	1113	15.0	(50.1)		13.4 (50.9)	16.5 (49.5)	22.1 (66.6)	16.4 (50.5)	8.3 (30.9)	15.8 (55.6)
	**Colombia**	1230	10.1	(39.8)		7.8 (31.2)	12.3 (46.5)	15.0 (41.5)	12.2 (42.7)	7.5 (29.8)	7.5 (43.7)
	**Costa Rica**	798	4.4	(27.9)		3.2 (28.4)	5.6 (27.3)	3.6 (25.4)	5.8 (36.3)	3.8 (20.9)	3.4 (17.6)
	**Ecuador**	800	11.6	(42.9)		12.4 (46.2)	10.9 (39.3)	17.1 (56.9)	13.4 (45.9)	9.3 (36.9)	6.0 (24.8)
	**Venezuela**	1132	5.1	(29.7)		3.4 (27.6)	6.7 (31.4)	4.4 (30.3)	5.9 (35.6)	4.7 (23.8)	4.4 (21.7)
**Red Meat**	**ELANS**	9218	61.3	(68.0)	<0.001	73.7 (76.5)	50.0 (56.9)	61.5 (65.4)	64.4 (70.3)	61.2 (69.1)	55.7 (63.5)
	**Argentina**	1266	79.1	(72.7)		93.1 (80.1)	67.4 (63.8)	80.5 (72.8)	80.4 (72.5)	79.5 (76.1)	75.7 (68.7)
	**Brazil**	2000	79.0	(82.2)		99.8 (94.7)	60.5 (63.7)	82.3 (79.3)	83.9 (86.9)	78.2 (82.0)	69.6 (74.5)
	**Chile**	879	45.7	(54.2)		57.5 (65.0)	34.7 (38.6)	37.8 (37.3)	49.9 (59.3)	49.9 (60.6)	38.6 (44.1)
	**Peru**	1113	26.0	(39.1)		31.7 (43.4)	20.9 (34.2)	21.3 (33.9)	28.7 (41.8)	24.7 (38.9)	25.4 (36.9)
	**Colombia**	1230	69.4	(65.8)		81.6 (71.5)	57.6 (57.4)	66.3 (60.6)	72.0 (66.7)	70.2 (63.7)	66.2 (69.1)
	**Costa Rica**	798	42.5	(51.9)		49.7 (58.2)	35.6 (43.9)	54.8 (66.3)	48.5 (53.4)	35.2 (45.9)	31.8 (39.4)
	**Ecuador**	800	63.8	(66.0)		72.9 (66.7)	54.8 (64.0)	60.5 (56.8)	70.3 (70.6)	60.7 (65.5)	56.6 (62.9)
	**Venezuela**	1132	59.7	(61.4)		70.1 (68.5)	49.7 (51.8)	73.4 (62.7)	61.6 (62.5)	58.4 (63.0)	46.7 (52.5)
**Processed Meat**	**ELANS**	9218	18.5	(34.1)	<0.001	22.2 (39.5)	15.1 (27.9)	20.3 (31.4)	21.3 (38.1)	17.2 (33.1)	13.9 (28.3)
	**Argentina**	1266	22.1	(35.3)		28.0 (41.5)	17.2 (28.3)	23.0 (33.1)	23.4 (34.9)	19.7 (33.7)	22.9 (38.9)
	**Brazil**	2000	22.2	(40.3)		26.1 (45.0)	18.7 (35.3)	24.6 (35.6)	25.8 (43.2)	19.9 (41.9)	17.6 (34.1)
	**Chile**	879	24.8	(43.9)		31.5 (54.0)	18.6 (30.5)	25.3 (34.4)	27.8 (56.8)	26.3 (38.9)	18.2 (29.7)
	**Peru**	1113	6.2	(14.0)		6.2 (14.2)	6.1 (13.7)	7.1 (14.4)	5.8 (13.1)	7.9 (16.7)	3.5 (10.1)
	**Colombia**	1230	18.2	(32.8)		22.2 (37.2)	14.2 (27.4)	26.5 (35.4)	22.0 (33.9)	16.9 (36.9)	9.8 (21.3)
	**Costa Rica**	798	20.8	(36.0)		26.2 (42.2)	15.5 (27.8)	23.0 (37.1)	27.3 (44.8)	17.1 (27.4)	11.5 (21.2)
	**Ecuador**	800	12.1	(27.5)		14.0 (32.1)	10.3 (21.9)	10.3 (16.6)	17.3 (36.9)	9.9 (20.2)	5.3 (15.7)
	**Venezuela**	1132	18.4	(25.6)		20.6 (28.6)	16.3 (22.2)	21.4 (29.2)	21.4 (28.0)	16.3 (21.7)	12.7 (21.3)
**Sugar-Sweetened Beverages: ready-to-drink**	**ELANS**	9218	313.9	(415.2)	<0.001	386.3 (474.0)	247.6 (339.4)	403.4 (455.6)	364.7 (444.6)	291.9 (399.6)	193.3 (309.4)
	**Argentina**	1266	712.6	(670.4)		896.4 (766.0)	560.6 (534.3)	951.1 (670.7)	825.6 (723.9)	667.2 (645.5)	472.3 (520.7)
	**Brazil**	2000	304.7	(342.7)		372.8 (381.4)	244.2 (291.3)	440.6 (388.9)	351.5 (352.3)	286.2 (338.5)	170.1 (241.0)
	**Chile**	879	262.3	(322.3)		358.3 (377.5)	172.5 (226.2)	298.8 (311.2)	333.7 (389.9)	251.9 (291.0)	145.7 (194.0)
	**Peru**	1113	196.8	(254.5)		250.8 (294.5)	148.9 (201.3)	212.0 (239.1)	206.5 (243.6)	202.0 (285.1)	152.6 (238.1)
	**Colombia**	1230	189.1	(263.1)		232.7 (302.0)	147.2 (211.3)	294.0 (399.2)	218.5 (266.3)	173.7 (231.4)	111.4 (166.9)
	**Costa Rica**	798	291.4	(340.1)		351.0 (385.0)	233.4 (278.0)	405.0 (319.0)	366.0 (388.0)	239.6 (303.7)	129.9 (206.2)
	**Ecuador**	800	192.8	(241.9)		255.0 (279.6)	131.6 (178.1)	216.2 (248.6)	242.2 (265.7)	151.9 (194.6)	122.0 (219.5)
	**Venezuela**	1132	276.7	(359.6)		317.5 (392.3)	238.0 (320.9)	351.5 (411.2)	342.6 (385.6)	228.3 (325.5)	145.7 (241.0)
**Sugar-Sweetened Beverages: homemade**	**ELANS**	9218	387.6	(418.0)	<0.001	407.2 (437.2)	369.6 (398.7)	341.3 (334.1)	381.4 (401.6)	413.8 (457.4)	329.4 (436.1)
	**Argentina**	1266	532.6	(637.8)		522.2 (627.1)	541.3 (646.8)	381.8 (353.3)	522.5 (554.2)	587.3 (753.1)	555.8 (696.8)
	**Brazil**	2000	249.1	(244.7)		266.0 (273.3)	234.1 (215.0)	267.9 (275.9)	230.5 (233.7)	272.5 (240.5)	237.7 (248.4)
	**Chile**	879	28.5	(64.3)		31.8 (72.7)	25.3 (55.1)	34.0 (72.3)	28.3 (66.4)	25.6 (50.9)	29.1 (71.0)
	**Peru**	1113	756.4	(429.3)		835.9 (471.4)	685.9 (374.6)	708.2 (372.6)	766.7 (439.2)	762.7 (460.7)	763.5 (400.4)
	**Colombia**	1230	319.4	(298.4)		330.7 (316.5)	308.4 (279.8)	280.4 (252.4)	304.6 (278.6)	322.8 (294.9)	356.4 (345.1)
	**Costa Rica**	798	426.7	(372.8)		476.9 (408.1)	377.8 (328.0)	287.7 (252.9)	392.1 (346.7)	540.8 (431.3)	437.7 (363.1)
	**Ecuador**	800	541.8	(326.7)		564.0 (351.8)	520.0 (298.8)	514.8 (286.2)	551.0 (355.8)	568.3 (349.5)	502.1 (298.8)
	**Venezuela**	1132	323.6	(300.0)		332.5 (312.2)	315.1 (287.8)	213.8 (209.7)	290.1 (293.9)	359.9 (308.6)	427.3 (319.3)

* Food Groups: food or beverages with demonstrated associations with cardiometabolic disease or any cause of mortality.

** Kruskal Wallis test. Data obtained from the ELANS by the average of two non-consecutive 24-HR.

The mean consumption of vegetables in total ELANS population was 86.7 g/day (62.1 g/day in Brazil—139.4 g/day in Ecuador). Vegetable intake tended to be higher in men and increased with age and SEL in most countries. However, Ecuador, Peru, Venezuela and Chile did not show a SEL-dependent trend in vegetable consumption.

Men reported higher intake of legumes than women, except for Venezuela where consumption was practically equal between genders. At high SEL, legumes/beans consumption tended to be lower in most countries, although only statistically significant different for Brazil. Consumption of nuts was very low (1.6 g/day) for the overall sample even though Colombians consumed 15-fold more nuts than Venezuelans (4.6 g/day vs 0.3 g/day, respectively). Regarding fish intake, the difference was almost 5-fold when comparing intake in Argentina (6.5 g/day) vs Peru (28.9 g/day) and Ecuador (30.3 g/day). Average whole grain intake in ELANS was 13.1 g/day with a less distinct difference between countries. The intake was higher at high SEL in most countries, with the exception of Peru and Colombia. Yogurt intake also showed a significant country-related difference (4.4 g/day in Costa Rica -20.9 g/day in Chile). This food group also exhibited a higher intake at high SEL, except for Argentina and Venezuela.

Mean consumption of red meat for the ELANS sample was 61.3 g/day (79 g/day in Argentina and Brazil—26 g/day in Peru). Red meat intake decreased with age and was higher among men in most countries (with the exception of Chile, Peru, and Colombia). Processed meat intake varied from 24.8 g/day in Chile to 6.2 g/day in Peru, and was higher among men in almost all countries, except for Peru where the intake was equivalent between genders.

Mean consumption of homemade SSB for the total ELANS sample was 387.6 g/day. Peruvians consumed almost 27-fold more homemade SSB than Chileans (756.4g/day and 28.5 g/d, respectively). The mean intake of ready-to-drink SSB was 313.9 g/day for the total ELANS sample (189.1 g/day in Colombia-712.6 g/day in Argentina). For both forms of SSB, men consumed more than women, with the exception of Argentina where intake by both genders were similar (522.2 vs 541.3g/day, respectively).

Comparison of the average dietary intake of the fruits and vegetables with the global recommendation (WHO) and minimum requirement evidenced by the literature to decrease the relative risk for NCDs are presented in Table 4.

**Table 4:** Prevalence of optimal consumption of the ten major foods groups in individuals residing in urban areas of eight Latin American countries.

Food Groups	Optimal consumption	ELANS n = 9218 (%)	Argentina n = 1266 (%)	Brazil n = 2000 (%)	Chile n = 879 (%)	Peru n = 1113 (%)	Colombia n = 1230 (%)	Costa Rica n = 798 (%)	Ecuador n = 800 (%)	Venezuela n = 1132 (%)
**Global reference (WHO)**										
Fruits and Vegetables	400 g/day^a^	7.2	6.9	5.6	11.5	10.6	4.8	8.9	11.3	2.1
**Minimum requirement evidenced by the literature to decrease the relative risk for NCDs**										
Fruits	200 g/day^h^	11.5	11.2	9.9	16.2	19.4	10.8	11.9	13.8	2.5
Vegetables	300 g/day^i^	2.4	2.5	2.2	3.0	0.5	1.4	4.9	5.8	1.0
Legumes/Beans	100 g/day^h,j^	13.1	1.0	13.3	6.3	7.7	15.4	41.7	22.4	7.7
Nuts and Seeds	10 g/day^h^	3.3	2.7	2.6	2.6	2.4	6.4	4.6	5.6	0.8
Whole Grains	100 g/day^h,i^	2.4	2.2	3.5	2.5	1.8	3.3	1.9	2.6	0.6
Fish and Seafood	200 g/day^i^	1.1	0.3	2.4	0.1	1.5	0.7	0.3	1.5	1.0
Yogurt	200 g/day^k^	1.4	2.7	1.6	1.5	1.6	0.8	0.5	1.1	0.6

* Data obtained from the ELANS by the average of two non-consecutive 24-HR. According to the proposed global, national and evidence-based/literature reference cut offs by ^a^ WHO Guidelines [46], and adapted from ^h^ Bechthold et al [5], ^i^ Schwingshackl et al [4], ^j^ Micha et al [8], ^k^ Wu et al [10].

When compared to the WHO’s recommendation for fruits and vegetables consumption (400 grams per day), it was observed that only 7.2% of the overall sample reached this recommendation, with the lowest proportion observed in Venezuela (2.1%). Regarding the dietary patterns related to a reduce risk of NCDs, among the overall sample legumes and fruits were the food groups whose intakes were closer to the recommendation, although much lower than expected (13.1% and 11.5%, respectively). For other major food groups such as vegetables, nuts, whole grains, fish and yogurt, only less than 3.5% of the sample met the optimal consumption level (Table 4).

Overall, 53.3% of the ELANS sample consumed fruits, with the highest prevalence observed in Chile (73.6%) and lowest percentage in Venezuela (24.5%) (Table 5). Interestingly, Peru was the country with the highest intake of fruits (232.10 ±212.9 g/day) among consumers.

**Table 5:** Comparison of dietary intake of the ten major foods groups (grams/day) in individuals residing in urban areas of Latin America, according to the country of living. Data corresponds to consumers* only.

Food Groups	Country	n	%	Mean g/day (SD)		p**	Percentiles of intake (g/day)						
							5	10	25	50	75	90	95
**Fruits**	**ELANS**	4915	53.3	204.3	(197.9)	<0.001	38.5	49.0	90.0	150.0	260.0	402.2	521.0
	**Argentina**	650	51.3	213.7	(163.2)		60.4	85.0	110.5	157.2	261.7	404.6	502.8
	**Brazil**	1119	56.0	180.0	(176.4)		26.0	40.0	80.0	135.0	225.0	366.6	510.0
	**Chile**	647	73.6	186.3	(167.8)		45.0	45.0	88.0	135.0	230.0	363.0	489.0
	**Peru**	784	70.4	232.1	(212.9)		44.0	66.0	92.0	172.0	288.0	458.5	594.0
	**Colombia**	592	48.1	215.7	(221.5)		39.6	60.0	100.0	157.9	270.0	412.0	520.0
	**Costa Rica**	394	49.4	205.4	(192.8)		23.6	42.0	82.0	143.0	282.0	429.0	534.0
	**Ecuador**	452	56.5	228.0	(269.7)		45.0	74.1	107.0	158.4	272.0	417.5	550.6
	**Venezuela**	277	24.5	178.5	(171.3)		20.0	34.7	81.4	137.5	215.5	345.0	450.0
**Vegetables**	**ELANS**	8541	92.7	108.1	(115.8)	<0.001	9.0	16.0	35.5	76.4	140.0	238.3	310.0
	**Argentina**	1176	92.9	109.0	(127.5)		12.0	20.0	40.0	74.0	135.0	229.7	317.0
	**Brazil**	1614	80.7	98.4	(140.1)		3.3	8.5	26.8	60.0	119.0	217.0	308.6
	**Chile**	839	95.5	138.6	(100.8)		24.6	37.0	61.3	120.0	185.0	273.1	340.0
	**Peru**	1111	99.8	92.2	(75.9)		7.8	15.1	37.1	72.8	127.5	197.0	241.7
	**Colombia**	1120	91.1	94.8	(104.4)		7.6	10.0	25.9	59.6	121.5	231.7	310.0
	**Costa Rica**	798	100.0	118.8	(144.0)		11.8	18.6	37.5	79.1	148.6	259.8	371.4
	**Ecuador**	799	99.9	144.9	(115.6)		13.3	22.8	59.3	120.5	200.9	303.7	369.9
	**Venezuela**	1084	95.8	87.9	(82.6)		10.6	16.9	29.6	65.4	117.6	188.8	245.5
**Legumes/Beans**	**ELANS**	5533	60.0	95.7	(103.0)	<0.001	7.1	12.0	32.0	64.0	120.0	208.7	286.9
	**Argentina**	164	13.0	58.1	(88.6)		1.5	4.3	16.0	32.0	62.0	115.5	223.2
	**Brazil**	1745	87.3	74.4	(58.1)		19.7	32.0	36.1	54.0	89.2	136.0	172.0
	**Chile**	397	45.2	81.9	(105.2)		5.0	5.0	13.0	50.0	116.1	175.0	277.2
	**Peru**	844	75.8	56.7	(70.8)		4.0	5.3	9.2	23.1	83.3	159.0	191.1
	**Colombia**	710	57.7	132.8	(148.8)		11.8	19.2	40.0	85.1	165.0	317.0	400.0
	**Costa Rica**	701	87.8	140.7	(113.3)		17.0	30.0	67.0	114.4	187.2	284.0	349.1
	**Ecuador**	594	74.3	118.9	(124.5)		11.2	13.0	28.8	80.0	179.7	275.0	359.4
	**Venezuela**	378	33.4	137.6	(132.0)		10.8	15.0	50.5	119.0	158.2	297.5	395.1
**Nuts and Seeds**	**ELANS**	771	8.4	35.0	(76.0)	<0.001	1.0	1.6	3.6	10.0	30.0	87.7	127.6
	**Argentina**	55	4.3	35.0	(44.7)		3.0	3.6	10.0	22.5	49.0	62.0	100.0
	**Brazil**	127	6.4	25.3	(39.1)		1.5	2.4	5.5	12.0	28.3	59.5	101.4
	**Chile**	51	5.8	19.3	(18.4)		1.6	3.1	7.0	13.5	30.0	45.0	76.2
	**Peru**	167	15.0	15.9	(71.0)		0.6	1.0	2.0	3.7	11.7	30.0	48.0
	**Colombia**	128	10.4	80.9	(124.5)		2.8	4.2	8.5	30.0	113.5	254.8	430.8
	**Costa Rica**	68	8.5	43.3	(55.0)		2.0	3.0	7.0	20.0	68.0	109.6	131.0
	**Ecuador**	154	19.3	28.5	(68.9)		0.6	1.2	2.3	5.0	20.0	50.0	140.0
	**Venezuela**	21	1.9	26.9	(49.2)		1.6	2.3	5.0	12.0	30.0	45.2	67.8
**Whole Grains**	**ELANS**	3345	36.3	56.1	(65.7)	<0.001	9.0	12.0	20.0	37.0	67.1	116.1	168.0
	**Argentina**	395	31.2	69.7	(66.5)		12.0	16.0	30.0	57.2	86.1	144.5	164.5
	**Brazil**	704	35.2	59.1	(68.1)		10.0	15.0	20.0	38.7	65.0	125.0	200.0
	**Chile**	245	27.9	59.6	(53.5)		7.6	12.0	20.0	40.0	80.0	140.0	174.5
	**Peru**	623	56.0	38.5	(50.3)		7.8	10.9	14.6	22.2	44.9	81.2	109.8
	**Colombia**	433	35.2	68.1	(83.2)		7.0	12.0	23.5	42.6	77.1	150.0	230.0
	**Costa Rica**	327	41.0	56.3	(55.1)		7.8	14.0	24.6	42.0	70.0	107.0	150.0
	**Ecuador**	250	31.3	62.7	(96.5)		7.5	10.0	15.0	40.0	62.5	130.0	250.0
	**Venezuela**	368	32.5	47.5	(40.2)		9.0	10.8	18.0	36.0	56.7	100.0	132.5
**Fish and Seafood**	**ELANS**	2427	26.3	121.2	(143.5)	<0.001	11.6	21.5	49.0	90.2	158.0	240.0	320.0
	**Argentina**	131	10.4	119.1	(111.5)		18.9	20.7	40.5	98.0	146.7	260.0	330.0
	**Brazil**	369	18.5	201.9	(302.2)		8.2	17.3	68.8	150.0	240.0	400.0	500.0
	**Chile**	197	22.4	101.0	(63.3)		20.0	36.7	60.0	90.0	126.0	163.0	240.0
	**Peru**	453	40.7	120.0	(90.3)		19.6	27.4	57.6	87.0	167.6	242.3	300.0
	**Colombia**	255	20.7	131.9	(96.9)		28.0	32.8	60.0	110.0	176.0	270.0	325.6
	**Costa Rica**	343	43.0	75.1	(74.1)		6.2	7.2	15.0	50.0	110.9	160.0	206.0
	**Ecuador**	402	50.3	95.1	(73.5)		26.0	30.0	41.0	80.8	130.0	178.8	220.4
	**Venezuela**	277	24.5	128.2	(93.1)		25.7	30.0	61.0	114.4	184.8	225.0	290.0
**Yogurt**	**ELANS**	888	9.6	214.1	(134.4)	<0.001	90.0	100.0	129.4	190.0	250.0	383.2	466.0
	**Argentina**	159	12.6	258.4	(168.3)		93.2	125.0	170.0	200.0	310.7	466.0	579.9
	**Brazil**	165	8.3	215.8	(128.7)		90.0	100.0	103.6	200.0	225.0	400.0	517.8
	**Chile**	172	19.6	167.8	(88.3)		120.0	124.3	125.0	129.4	181.2	258.9	375.0
	**Peru**	132	11.9	227.3	(150.1)		74.0	100.0	130.0	196.0	289.0	366.0	500.0
	**Colombia**	102	8.3	217.6	(101.8)		93.2	103.6	155.3	206.1	212.3	331.4	410.0
	**Costa Rica**	31	3.9	208.7	(158.7)		85.0	100.0	125.0	178.0	229.0	300.0	403.0
	**Ecuador**	72	9.0	221.4	(109.0)		103.6	124.3	155.3	207.1	248.5	310.7	310.7
	**Venezuela**	55	4.9	191.9	(153.8)		15.0	15.0	125.0	155.3	258.9	414.2	507.4
**Red Meat**	**ELANS**	6740	73.1	118.2	(91.6)	<0.001	19.0	29.5	56.0	100.0	155.9	235.9	299.1
	**Argentina**	1042	82.3	129.7	(95.2)		20.0	33.1	63.0	110.0	172.2	247.8	303.0
	**Brazil**	1535	76.8	141.7	(109.4)		20.0	35.0	70.0	105.4	186.0	300.0	350.0
	**Chile**	629	71.6	95.1	(80.4)		21.0	28.5	42.8	80.0	100.0	192.0	250.0
	**Peru**	561	50.4	78.7	(60.6)		14.7	19.0	37.0	60.0	100.7	153.5	196.6
	**Colombia**	982	79.8	117.7	(82.3)		24.1	37.5	60.0	101.8	155.9	230.0	259.9
	**Costa Rica**	570	71.4	86.4	(77.5)		12.5	17.6	31.5	63.0	117.0	180.0	240.0
	**Ecuador**	599	74.9	122.0	(83.9)		26.3	32.9	70.2	100.0	160.0	240.0	299.1
	**Venezuela**	822	72.6	118.7	(79.7)		20.0	35.0	60.0	115.0	156.0	201.6	260.0
**Processed Meats**	**ELANS**	4560	49.5	58.8	(63.9)	<0.001	4.9	9.8	20.0	40.0	75.0	125.0	180.0
	**Argentina**	644	50.9	69.8	(63.4)		8.5	13.3	25.0	50.0	95.0	150.0	200.0
	**Brazil**	985	49.3	70.7	(76.6)		9.8	15.0	20.0	46.3	90.0	160.0	226.0
	**Chile**	516	58.7	62.0	(68.8)		10.0	15.0	20.5	40.0	80.0	135.0	188.0
	**Peru**	304	27.3	40.9	(33.3)		8.0	9.0	16.0	32.0	61.0	83.0	109.0
	**Colombia**	677	55.0	53.0	(61.3)		3.7	5.2	11.8	34.4	69.4	118.0	160.0
	**Costa Rica**	468	58.7	53.8	(67.4)		4.0	6.2	13.6	30.0	68.0	122.0	164.0
	**Ecuador**	273	34.1	61.4	(66.2)		15.0	15.0	30.0	50.0	65.0	120.0	180.0
	**Venezuela**	693	61.2	45.0	(39.7)		1.4	1.8	25.0	33.0	51.4	95.6	119.0
**Sugar-Sweetened Beverages: ready-to-drink**	**ELANS**	6386	69.3	596.4	(524.6)	<0.001	138.0	200.2	281.0	450.0	739.1	1196.5	1557.2
	**Argentina**	1060	83.7	977.9	(764.2)		200.5	285.5	450.9	794.2	1260.7	1920.5	2385.4
	**Brazil**	1431	71.6	558.1	(405.7)		187.5	207.6	300.0	421.7	700.0	1038.1	1349.6
	**Chile**	606	68.9	500.2	(385.9)		140.1	207.6	259.5	364.5	624.9	947.7	1245.7
	**Peru**	685	61.6	456.3	(323.8)		131.5	168.5	225.5	409.5	504.0	930.0	1122.5
	**Colombia**	759	61.7	443.8	(394.1)		94.3	155.7	220.7	353.7	600.6	811.3	1029.8
	**Costa Rica**	580	72.7	523.2	(436.0)		82.7	154.3	250.0	380.0	664.0	1065.0	1347.4
	**Ecuador**	510	63.8	433.3	(306.4)		77.9	155.7	293.0	312.5	589.1	778.6	961.6
	**Venezuela**	755	66.7	547.2	(466.7)		30.1	222.2	311.4	419.3	623.9	1038.1	1308.0
**Sugar-Sweetened Beverages: homemade**	**ELANS**	7650	83.0	533.6	(479.2)	<0.001	24.8	64.0	225.0	408.9	729.3	1084.6	1406.2
	**Argentina**	948	74.9	817.8	(729.5)		204.2	230.2	315.3	593.0	1036.9	1611.3	2101.8
	**Brazil**	1564	78.2	368.3	(287.1)		48.0	83.1	196.2	302.7	494.9	722.0	915.7
	**Chile**	540	61.4	54.7	(112.1)		6.0	8.0	12.4	24.0	36.0	98.0	275.8
	**Peru**	1079	97.0	826.2	(487.6)		262.0	296.3	500.0	752.6	1041.6	1424.5	1682.4
	**Colombia**	1089	88.5	427.7	(369.1)		20.0	43.0	180.5	339.7	584.4	876.1	1145.8
	**Costa Rica**	663	83.1	591.7	(401.7)		180.0	227.0	292.0	509.0	777.0	1076.0	1338.0
	**Ecuador**	776	97.0	617.9	(384.5)		194.1	251.4	313.2	554.9	807.9	1099.5	1353.3
	**Venezuela**	991	87.5	433.0	(338.0)		81.3	114.9	195.0	378.6	567.9	818.1	1094.7

* Consumers were considered those individuals reporting positive intakes in one or two 24-HR

** Kruskal Wallis test. Data obtained from the ELANS, considering the consumption in at least one 24-HR.

The prevalence of vegetable consumption was higher than fruits. A higher percentage of the ELANS sample consumed vegetables (92.7%), ranging from 80.7% in Brazil to 100% in Costa Rica.Legumes (or beans) were highly consumed in Costa Rica (87.8% of the sample) and Brazil (87.3%). The consumption of nuts and seeds was very low among countries (8.4%). In the overall sample, 36.3% consumed whole grains, with the highest percentage in Peru (56.0%). Just 26.3% of the ELANS population consumed fish and/or seafood, highlighting Ecuador, where half of the sample consumed fish (50%) versus 10.4% of Argentina’s sample. Yogurt consumption was low among ELANS countries (9.6%), and Chile was the country with the highest prevalence of consumption (almost 20%).

Red meat consumption was highly prevalent among all countries (73.1%), with a higher consumption in Argentina (82.3% of the sample) followed by Brazil (76.8%).

SSB analyses considered the sub-groups ready-to-drink and homemade SSB. Around 70% of ELANS population consumed ready-to-drink SSB, and Argentina had the highest prevalence of consumption (83.7%). Around 83% of the ELANS population and almost the entire sample of Ecuador and Peru (97%) consumed homemade SSB.

## Discussion

In this first Latin American study, the consumption of major food groups negatively or positively associated with NCDs was assessed and compared with current international intake recommendations. Overall, mean intakes of healthy food groups such as fruits, vegetables, nuts/seeds, whole grains, fish and yogurt were markedly below current recommendations or optimal intakes and had a SEL-dependent trend in consumption. Our results also demonstrated that among consumers, the most frequently consumed foods with positive or negative association with NCDs were vegetables (93%) and red meats (73%), respectively.

### Foods with protective associations against clinical disease outcomes

#### Fruits and vegetables

Fruits and vegetables are widely available within Latin America. This world region has ample crops with variety and richness in nutrients. Under these conditions, we expected a higher intake of fruits and vegetables among the countries participating in the ELANS. Only 7.2% of the participants met the WHO intake recommendation for fruits and vegetables (400 g/day [46]), with wide differences between countries and SEL. Ecuador, Chile, and Peru showed a higher intake of fruits while only 2.4% of the Venezuelan sample attained this cut-off. Consumption levels are significantly lower than those observed in North America where 13% and 24% of US citizens meet vegetables and fruits intake recommendations, respectively [47].

Even among those who consume fruits and vegetables, the mean intake remained below existing recommendations. A trend on increasing intake with age was observed for fruits and vegetables in almost all countries: older people consumed 50% more fruits than younger ones (95.7 vs 64.4 g/day) and 23% more vegetables. These findings are consistent with previous reports. A study with a Spanish population [48] showed lower fruits and vegetables intake from children to young adults and then it increased to subjects 75 year old. In Germany, fruits intake increased in both men and women up to the age of 60–69 years [49].

In contrast to previous national surveys, we did not find clear differences in fruits and vegetables consumption by gender.

The association between SEL and fruits and vegetables consumption has been previously evaluated. For example, the Canadians aged 12 and older who consumed fruits and vegetables at least five times daily was highest in the highest income quintile [50]. The proportion of men and women in Germany who consume at least three portions every day also increases with SEL [49]. Furthermore, women of higher economic status ate significantly more fruits (P<0.05) and more fruits and vegetables combined (P<0.05) [51]. In addition, higher fruits and vegetables intake scores were also observed in neighborhoods with a higher density of healthy food outlets and higher income [52]. These studies support our findings showing that both fruits and vegetables are consumed in greater amounts by people from high compared to low SEL in Latin American countries participating in ELANS except for Ecuador, where vegetables distribution consumption seems to be more homogeneous regardless of the socioeconomic level.

It is important to highlight the overall distribution of this representative sample of 8 Latin American countries, in which 52% (n = 4796) of the population are from the low SEL group. UK researchers who had studied the relationship between quintiles of economic distribution and access to fruits and vegetables have identified some strong determinants showing that individuals in the lowest quartile of food expenditure, low income and low education consumed less fruit and vegetables, a result which indicates that the association between the costs of purchasing foods and fruits and vegetable intake was stronger for less educated and lower income groups. In other words, socioeconomic differences in fruits and vegetables intake were heightened among individuals who consumed low-cost diets [53]. Such socioeconomic inequalities in food consumption among those consuming low-cost diets indicate the need to address food costs when designing strategies to promote healthy diets.

In Venezuela, the ongoing economic crisis may have affected the diet and limited access to many nutritious foods including fruits and vegetables. A better understanding of additional barriers at national levels may allow the implementation of public policies to increase fruits and vegetables consumption.

#### Nuts and seeds

Only 8.4% of our study population reported any consumption of nuts and/or seeds. While the highest percentage of this food group intake was found in Ecuador. Comparisons with other world regions are limited due to methodological differences in data collection. Most studies analyze nuts and seeds consumption based on a weekly frequency, with very few studies—including ELANS—reporting consumption exclusively by 24-HR. For instance, New Zealanders reported an average intake of nuts of 5.2 g/day and 35.0 g/day of nuts and seeds [54]. The US National Health and Nutrition Examination Survey has reported that almost 40% of adults consumed nuts on a given day with a higher prevalence of intake in non-Hispanic whites (43.6%) compared to non-Hispanic blacks (23.7%) [55].

As for other healthy food groups, it is difficult to assess the relationship between healthy nuts intake and affordability. Nuts are a high-cost item of a healthy dietary pattern (exempting peanuts) [56]. Thus, all countries surveyed in ELANS–except for Colombia and Ecuador–showed higher consumption at higher SEL but it must be considered that a small proportion of the population reported nuts and seeds consumption.

With respect to the attainment of current intake recommendations, only 3% of the Latin American population recruited in this study exceeded the consumption of 10 g/day, a cut-off point associated with the prevention of cardiovascular diseases.

#### Yogurt

While the positive relationship between overall dairy products consumption and CVD and metabolic syndrome remains controversial, yogurt consumption seems to exhibit more consistent beneficial effects on human health [57]. Indeed, observational studies and randomized trials have associated yogurt consumption with a reduced risk of weight gain and obesity as well as CVD [58]. However, there is no consensus on its recommended intake.

Prevalence of yogurt consumption in Latin America was only 9.3%. Yogurt consumption is determined by culture, availability as well as food budget. As is the case in European countries with habitual yogurt consumption [59], Argentina and Chile, the most southern Latin American countries are the leaders of yogurt intake within the region. In addition, the younger age group was more likely to consume yogurt than older subjects and its consumption is more prevalent within the high socioeconomic level in almost all Latin American countries. In Argentina, yogurt intake is also associated with the consumption of milk and other dairy products and directly correlates with calcium intake [60].

#### Whole grains and legumes

Whole grains are the main dietary source of fibers and their consumption is associated with lower incidence of many chronic diseases (such as colon cancer, type 2 diabetes, and others) [9, 40, 61]. For example, 3 servings of whole grains per day (45 g/day) were associated with 20% lower risk of type 2 diabetes compared to consuming half a serving (7.5 g/day) [62].

In ELANS, the average whole grain intake was 13.5 g/day for consumers, with minimal differences between countries, but a clear higher consumption in high versus low SEL. Other countries report similar consumption, including 14.4 g/day in French adults [63]. In our study, only one-third of men and women consumed whole grain, similar to the French study with a third of the respondents reporting consuming whole grain [63]. Other countries with more developed whole grain intake culture, such as England, have reported a mean intake of 26.2 g/day[64].

Legumes are also a good source of fibers and micronutrients [65] and wider differences were seen between countries. Remarkably, 41% of the Costa Rican sample consumed 100 g/day or more, a cut-off value associated with positive health outcomes, in comparison with only 1% in Argentina. The highest consumption was observed in Central American countries and Brazil. Typical dishes in those countries include legumes and are part of the daily breakfast, while in southern countries typical breakfast is more European-based style.

It is interesting to note that, in contrast to other healthy food groups, high legumes intake was associated with lower SEL. Both culture and affordability of this food group seemed to explain this behavior. In contrast, in Ecuador and Costa Rica where legumes are consumed by a vast majority of the population, this food group intake was not different among the SEL groups.

#### Fish and seafood

The Latin American region is surrounded by sea and all eight ELANS countries have access to coastlines and sea products. Despite this geographic advantage, fish and seafood consumption is uneven with wide country variations: Ecuador and Peru reported intakes of 30 g/day, while mean consumption in Argentina was 6.5 g/day. European countries have reported higher fish and seafood intakes of 26.9 g/day for men across the European countries, with 68.7 g/day reported for Spanish men, and 73.2 g/day for Norwegian women [66].

Only one-fourth of the ELANS population declared to be fish consumers. Subjects from Ecuador, Peru and Costa Rica were the highest consumers (50, 40 and 42%, respectively). Mean daily intake by people who ate fish and seafood was 121 g/day. No differences by gender or age groups were found for the intake of this food group in ELANS.

A smaller percentage of individuals from lower income–compared to higher-income–households as well as disadvantaged socio-economic indicators in education and occupation reported consuming seafood, fish or shellfish [67]. Interestingly, some Latin American countries, such as Peru, Ecuador, and Venezuela, seemed to exhibit an inverse association between sea products intake and SEL, whereas consumption of this food group is favored by higher SEL, in Argentina, Colombia and Costa Rica.

With regards to the current recommendations for seafood [4], only 1.1% of the ELANS population reported consuming equal or above 200 grams/day proposed as associated with beneficial effects on cardiometabolic outcomes.

### Foods with negative association with health outcomes

#### Sugar-sweetened beverages (SSB)

Sugar-sweetened beverages, including homemade beverages prepared with added sugars such as coffee, tea, mate, and juices, are consumed worldwide. The negative effects of SSB intake on human health involve obesity, metabolic syndrome, diabetes mellitus, cardiovascular diseases, and cancers [68].

In this study, SSB were analyzed divided into two groups: ready-to-drink products including soft drinks, artificial juices and other natural juices with added sugars; and homemade drinks, including various juices and infusions and other homemade beverages enriched with added sugars. Analyzing the overall ELANS sample, we found a high percentage of SSB consumption: 69.3% reported consumption of ready-to-drink SSB and 82.9% of homemade SSB with significant differences by country, age, gender and SEL. Mean intake consumption was 314 and 388 g/day for ready-to-drink and homemade SSB, respectively. Argentina and Peru were the leading consumers of SSB (ready-to-drink + homemade) with a mean intake of 1,245 and 953 g/day, respectively, followed by Ecuador, Costa Rica and Venezuela. These findings indicate much higher proportion of SSB intake(SSBs ≥1 time/day) in LA than other world regions [69].

In the current study it was observed a decrease of ready-to-drink SSB consumption in Latin America over the life course: adults and older adults consumed less ready-to-drink SSB than younger age groups in all ELANS countries. Regarding homemade beverages, this age-related pattern was not consistent within the study sample, including some countries such as Argentina, Venezuela and Costa Rica, where intake of the SSB beverages consistently increases with age. With minimal differences found by country, the whole ELANS sample exhibited similar intake of both ready-to-drink and homemade SSB by SEL.

This regional pattern of SSB consumption may be related to economic and cultural traditions as well as food Westernization in Latin America. Sugar is a major commodity of the region and it is the main component of many local traditional dishes, liquid foods, and beverages, many of them inherited from the indigenous culture before colonization [70, 71]. Westernization of foods habits was associated with the increased availability of sugar-enriched foods and beverages. Thus, we hypothesize that this high consumption of SSB observed in the Latin America region developed from historical habits of local and traditional homemade SSB that have remained alive together with the rapid incorporation of processed foods and drinks. However, it has been observed that the contribution of homemade SSB to the total SSB varied between countries, ranging from 79% in Peru to 43% in Argentina (Homemade SSB/Total SSB x100%). Interestingly, Mexico is one of the Latin America countries in which SSB consumption has become a public health concern [72].

Our findings substantiate an urgent need for further understanding of main foods and beverages sources of total sugar intake within the region as well as to create public health strategies to diminish SSB consumption considering the ongoing epidemics of NCD in Latin America [73]. For instance, some studies have demonstrated a positive impact of the replacement of SSB consumption by water intake on obesity [74].

#### Red and processed meats

Reduced consumption of red and processed meats is not only directly associated with a lower risk of some types of cancers [75, 76] but also linked to a reduced risk of cardiovascular disease, type 2 diabetes, and obesity [77]. In addition, major concerns of public policy on environmental sustainability [78] have been related to these food products. However, red meat remains an important source of high-quality protein, zinc, B12 vitamin and iron for minorities and vulnerable populations [79].

The vast majority of the Latin American countries included in ELANS reported consuming red meat. Indeed, red meat was the second most consumed food group (73% reported intake) within the region after vegetables, fluctuating from 82% for Argentinians to 50% for Peruvians. Average absolute intake was 79.5 g of red and processed meat/day with socio-demographic disparities by age, gender and SEL. This mean value is somewhat lower than findings from previous surveys that reported a mean intake of 113 g/day in Sao Paulo/Brazil, where red and processed meat consumption was found in 75% of the sample [80]. In the US, the NHANES 2004 reported an intake of 93 g/day (69.8 g/d for red meat + 23.2 g/d for processed meat) for this food group for the total population [81].

Consistent with other studies, men from the ELANS sample reported red and processed meat consumption 50% higher than women, a pattern that was similar for all countries. Interestingly, red meat seems to be equally consumed by all socioeconomic strata regardless of the absolute differences between specific countries. Thus, red meat seems to be a rather uniform source of energy, proteins, and micronutrients in Latin America despite its negative health effects. In contrast, processed meats showed a trend to higher consumption at low SEL, particularly in Argentina and Chile.

Scientific debate remains on the daily/weekly intake of red meat that shows no negative health implications. One difference that characterized LA in comparison with US is that red meat is frequently cooked in the saucepan as part of combined dishes, or grilled in a slow cooker. However, common agreement suggests that limiting red and processed meat consumption would benefit health [77].

### Strengths and limitations

ELANS methodology of food intake data collection was very detailed, aiming to evaluate the amount consumed of each food and providing more accurate information compared to approaches based on food frequency questionnaires (FFQ). The two 24-h recall were conducted on different weekdays, including weekends, and different months of the year, thereby reducing inter-individual variability [82]. In order to further reduce methodological bias, adjustment of dietary data was performed by the Multiple Source Method, a statistical model in which data were adjusted for intrapersonal variability to estimate the usual intake of each nutrient in each individual [34, 83]. For the food groups, the daily intake of each participant was estimated considering the mean consumption from both records. In addition, dietary data were used in a quantitative model to estimate the amount consumed through linear followed by logistic regression with random effects. Furthermore, our large sample size is a strength that attenuates methodological biases due to limited data collection.

The 24-h recall method is rapid to apply and requires only short-time memory, which contributes to better respondent adherence and data quality [84, 85]. However, some limitations remain such as misreporting due to interviewees' bias and forgetfulness [86, 87]. In order to minimize such bias, the Multiple Pass Method was used to assist the interviewee in recalling and detailing his/her food report by five steps, including quick list, forgotten food list, time and occasion, detailed cycle, and final review probe [33, 88].

An important limitation of this study is the range of assumed “optimal consumption” which was based on cut off values according to a global reference [46, 89] and/or based on meta-analysis evidence for risk of CVD and/or mortality [4, 5, 8, 10, 14]. Another limitation is that the ELANS sample is composed only by the urban population of each country and therefore the findings cannot be extrapolated to the entire population.

The assessment of the dietary intake according to food groups required adapting the reality of the local population with regards to the food group composition, as well as to the cut off points. In this step it was necessary to take into account both the population's habits and culture [48, 90]. In addition, the absence of a FFQ that could differentiate the real consumers from non-consumers for the food groups analyzed in this study has limited the use of methods of estimation of the usual food intake that are capable of eliminating the intra-individual variability of intake such as NCI and/or MSM [91, 92]. The authors have chosen to report the mean intake of two 24-HR and not only the information reported on the first 24-HR, aiming this way, to reduce this underestimation of the food intake, especially for those food groups where the frequency of intake is low (e.g nuts, yogurt and fish).

Consistent with current trends in nutritional research, this study evaluated diet quality based on foods or food groups -instead of nutrients- in a representative sample of the urban population from Latin America. Our findings indicate that diet intake quality is deficient for all food groups, suggesting a high risk for chronic diseases and mortality in this world region in the upcoming decades. These data provide relevant and up-to-date information to design, implement, and evaluate national guidelines and recommendations depending on current dietary practices and intake patterns in each country that participated in ELANS. Improving this suboptimal diet should be one of the most critical tasks to be developed in Latin America in order to attenuate the ongoing transition towards NCDs, chronic disability, and decreased life expectancy.

The current study shows that in Latin America, there is a huge distance between the intake and the recommendation for the food groups commonly related to chronic diseases regardless of the country. The low percentage of individuals with a minimum of 1 to a maximum of 11% in fish and vegetables consumption, respectively, present a challenge for meeting thresholds considered in prevention of NCD. Health food groups tend to be more consumed in high SEL, and by older people. Vegetables and red meat are the most consumed food groups in the region, although the amounts of the first group are below the recommendations and the latter is overconsumed in most of the countries.

## Supporting information

S1 Text Food groups and beverages related to NCDs. Scientific evidence that supports the association between the ten foods groups (fruits; vegetables; legumes/beans; nuts and seeds; whole grains products; fish and seafood; yogurt; red meat; processed meats; sugar-sweetened beverages (ready-to-drink and homemade)) and NCD risk.
(DOCX)
