# Supplementary material for: Latin American consumption of major food groups: Results from the ELANS study
Source: PLoS One. 2019 Dec 26;14(12):e0225101. doi: 10.1371/journal.pone.0225101 (PMC6932811; doi:10.1371/journal.pone.0225101)
Supplement: S1 Text — Scientific evidence that supports the association between the ten foods groups (fruits; vegetables; legumes/beans; nuts and seeds; whole grains products; fish and seafood; yogurt; red meat; processed meats; sugar-sweetened beverages (ready-to-drink and homemade)) and NCD risk. (DOCX) [file pone.0225101.s001.docx]

We aimed to identify the latest scientific evidence on specific foods groups and beverages identified to be related to NCDs risk including CVD, diabetes, obesity, cancer, and all-cause mortality. Search criteria included PubMed-cited publications, meta-analyses, and publication dates within the last 5 years [1-26]. Scientific evidence that supports the association between specific foods groups and NCD risk is briefly described as follows.

*Fruits and Vegetables*

Some cancers, stroke and CVD can be reduced with an optimal consumption of fruits and vegetables. According to WHO [27], the adequate consumption of fruits and vegetables is able to reduce overall mortality and is a dietary factor to prevent CVD. The literature provides meta-analysis based evidence supporting risk reduction of CVD, hypertension, type 2 diabetes, coronary heart disease, stroke, heart failure, and cancer. In addition, total mortality (including non-cardiovascular mortality) is decreased with high consumption of fruits and vegetables [1-3, 9, 21, 28-30].

*Legumes/Beans*

Legumes –also called beans in some regions– show a healthy nutritional profile due to high mineral content, good source of vegetal protein, and fiber composition. A complete literature review together with meta-analyses and cohort data reports support a positive association between an high legume consumption and health benefits, such as risk reduction of heart ischemic disease, coronary heart disease, type 2 diabetes as well as all-cause mortality and non-cardiovascular mortality [1, 2, 23, 28, 31].

*Nuts and Seeds*

Nuts and seeds have a very peculiar nutritional composition with more than half weight constituted by lipids, predominantly made of monounsaturated and polyunsaturated fatty acids [32]. The lipid profile of nuts may be responsible of their protective effect on CVD [33]. Meta-analyses, cohort, and interventional studies provide evidence that ideal consumption of nuts and seeds is inversely associated with CVD, coronary heart disease, type 2 diabetes, total cancer, all-cause mortality, and mortality caused by respiratory diseases [1, 2, 7, 34, 35].

*Whole grains*

Recently, different meta-analyses reported risk reduction of NCDs (CVD, coronary heart disease, stroke, type 2 diabetes, total cancer, colorectal, pancreatic, and gastric cancers, all-cause mortality, cardiovascular and total cancer mortality, and death from respiratory, infectious, and nervous system diseases) associated with high consumption of whole grains [1, 2, 6, 8, 13, 15, 17].

*Fish and Seafood*

Unsaturated (mono- or poly-) fatty acids are considered protective nutrients for cardiovascular health based on evidence showing that omega-3 polyunsaturated fatty acids (ω-3 PUFAs) intake is strongly associated with CVD prevention [36-40] . However, the effects of concentrated ω-3 PUFAs administered as a supplement may be different from omega-3 provided by marine foods (fish and seafood) [39]. Also, fish or seafood consumption are sources of additional nutrients (e.g., taurine) that often have low intake prevalence among populations [38, 41]. Literature provides evidence that fish or seafood intake can prevent cardiovascular diseases, such as coronary heart disease, stroke, heart failure, acute coronary syndrome, and reduce risk of cardiovascular and all-cause mortality [1, 2, 25, 26, 40, 42].

*Yogurt*

Milk and dairy products are foods with positive nutritional properties –included in a unique food matrix- that provide benefits to health [43]. Milk-derived products, such as cheeses and yogurts, contain fermented milk –with different fat and protein content– together with bacteria determining unique characteristics on each dairy product and making difficult the assessment of the impact of these foods on health outcomes in meta-analyses. Indeed, the literature shows significant inconsistencies, with neutral and/or favorable outcomes relating dairy consumption and cardiovascular-related events [4, 5, 12, 18, 44]. In this context, the health benefits of yogurt intake seem to be more evident and promising [11, 45, 46].

*Red Meat*

Red meat consumption is frequently analyzed including unprocessed and processed formulations together. There is a controversial association between intake of this food group and health/disease. Red meat is an important source of essential nutrients, such as proteins, iron, and B vitamins. Evidence suggests that moderate consumption of red meat does not affect CVD risk factors, such as blood cholesterol and triglycerides and blood pressure, and is not associated with higher risk of type 2 diabetes, coronary heart disease, stroke, and neither increased mortality (all-cause, cardiovascular and cancer) [1, 2, 16, 19, 22, 47]. However, there is a positive association between risk of mortality and unprocessed red meat intake [1, 2, 19].

*Processed Meats*

Recent investigations have established a correlation between processed meat consumption and NCDs (e.g., coronary heart disease, stroke, and type 2 diabetes) risk and mortality (all-cause, cancer, and cardiovascular mortality) [1, 2, 19, 22, 47]. This association was corroborated in the literature by meta-analyses.

*Sugar-Sweetened Beverages*

WHO recognizes that a diet with high levels of free sugars, mainly those that come from sugar-sweetened beverages (SSB) (both ready-to-drink and homemade), is associated with a higher risk of NCDs and obesity [48]. Meta-analyses have linked SSB consumption with higher risk of CVD and cancer mortality, even though type 2 diabetes seems to be the main NCD outcome related to this food grouo [1, 14, 20, 24, 49, 50].

References

1. Bechthold A, Boeing H, Schwedhelm C, Hoffmann G, Knuppel S, Iqbal K, et al. Food groups and risk of coronary heart disease, stroke and heart failure: A systematic review and dose-response meta-analysis of prospective studies. Crit Rev Food Sci Nutr. 2017:1-20. doi: 10.1080/10408398.2017.1392288. PubMed PMID: 29039970.

2. Schwingshackl L, Schwedhelm C, Hoffmann G, Lampousi AM, Knuppel S, Iqbal K, et al. Food groups and risk of all-cause mortality: a systematic review and meta-analysis of prospective studies. Am J Clin Nutr. 2017;105(6):1462-73. doi: 10.3945/ajcn.117.153148. PubMed PMID: 28446499.

3. Schwingshackl L, Hoffmann G, Lampousi AM, Knuppel S, Iqbal K, Schwedhelm C, et al. Food groups and risk of type 2 diabetes mellitus: a systematic review and meta-analysis of prospective studies. Eur J Epidemiol. 2017;32(5):363-75. doi: 10.1007/s10654-017-0246-y. PubMed PMID: 28397016; PubMed Central PMCID: PMCPMC5506108.

4. Gholami F, Khoramdad M, Esmailnasab N, Moradi G, Nouri B, Safiri S, et al. The effect of dairy consumption on the prevention of cardiovascular diseases: A meta-analysis of prospective studies. J Cardiovasc Thorac Res. 2017;9(1):1-11. doi: 10.15171/jcvtr.2017.01. PubMed PMID: 28451082; PubMed Central PMCID: PMCPMC5402021.

5. Guo J, Astrup A, Lovegrove JA, Gijsbers L, Givens DI, Soedamah-Muthu SS. Milk and dairy consumption and risk of cardiovascular diseases and all-cause mortality: dose-response meta-analysis of prospective cohort studies. Eur J Epidemiol. 2017;32(4):269-87. doi: 10.1007/s10654-017-0243-1. PubMed PMID: 28374228; PubMed Central PMCID: PMCPMC5437143.

6. McRae MP. Health Benefits of Dietary Whole Grains: An Umbrella Review of Meta-analyses. J Chiropr Med. 2017;16(1):10-8. doi: 10.1016/j.jcm.2016.08.008. PubMed PMID: 28228693; PubMed Central PMCID: PMCPMC5310957.

7. Aune D, Keum N, Giovannucci E, Fadnes LT, Boffetta P, Greenwood DC, et al. Nut consumption and risk of cardiovascular disease, total cancer, all-cause and cause-specific mortality: a systematic review and dose-response meta-analysis of prospective studies. BMC Med. 2016;14(1):207. doi: 10.1186/s12916-016-0730-3. PubMed PMID: 27916000; PubMed Central PMCID: PMCPMC5137221.

8. Aune D, Keum N, Giovannucci E, Fadnes LT, Boffetta P, Greenwood DC, et al. Whole grain consumption and risk of cardiovascular disease, cancer, and all cause and cause specific mortality: systematic review and dose-response meta-analysis of prospective studies. BMJ. 2016;353:i2716. doi: 10.1136/bmj.i2716. PubMed PMID: 27301975; PubMed Central PMCID: PMCPMC4908315.

9. Aune D, Giovannucci E, Boffetta P, Fadnes LT, Keum N, Norat T, et al. Fruit and vegetable intake and the risk of cardiovascular disease, total cancer and all-cause mortality-a systematic review and dose-response meta-analysis of prospective studies. Int J Epidemiol. 2017;46(3):1029-56. doi: 10.1093/ije/dyw319. PubMed PMID: 28338764.

10. Micha R, Khatibzadeh S, Shi P, Andrews KG, Engell RE, Mozaffarian D, et al. Global, regional and national consumption of major food groups in 1990 and 2010: a systematic analysis including 266 country-specific nutrition surveys worldwide. BMJ Open. 2015;5(9):e008705. doi: 10.1136/bmjopen-2015-008705. PubMed PMID: 26408285; PubMed Central PMCID: PMCPMC4593162.

11. Micha R, Shulkin ML, Penalvo JL, Khatibzadeh S, Singh GM, Rao M, et al. Etiologic effects and optimal intakes of foods and nutrients for risk of cardiovascular diseases and diabetes: Systematic reviews and meta-analyses from the Nutrition and Chronic Diseases Expert Group (NutriCoDE). PLoS One. 2017;12(4):e0175149. doi: 10.1371/journal.pone.0175149. PubMed PMID: 28448503; PubMed Central PMCID: PMCPMC5407851.

12. Alexander DD, Bylsma LC, Vargas AJ, Cohen SS, Doucette A, Mohamed M, et al. Dairy consumption and CVD: a systematic review and meta-analysis. Br J Nutr. 2016;115(4):737-50. doi: 10.1017/S0007114515005000. PubMed PMID: 26786887.

13. Benisi-Kohansal S, Saneei P, Salehi-Marzijarani M, Larijani B, Esmaillzadeh A. Whole-Grain Intake and Mortality from All Causes, Cardiovascular Disease, and Cancer: A Systematic Review and Dose-Response Meta-Analysis of Prospective Cohort Studies. Adv Nutr. 2016;7(6):1052-65. doi: 10.3945/an.115.011635. PubMed PMID: 28140323; PubMed Central PMCID: PMCPMC5105035.

14. Imamura F, O'Connor L, Ye Z, Mursu J, Hayashino Y, Bhupathiraju SN, et al. Consumption of sugar sweetened beverages, artificially sweetened beverages, and fruit juice and incidence of type 2 diabetes: systematic review, meta-analysis, and estimation of population attributable fraction. Br J Sports Med. 2016;50(8):496-504. doi: 10.1136/bjsports-2016-h3576rep. PubMed PMID: 27044603; PubMed Central PMCID: PMCPMC4853528.

15. Li B, Zhang G, Tan M, Zhao L, Jin L, Tang X, et al. Consumption of whole grains in relation to mortality from all causes, cardiovascular disease, and diabetes: Dose-response meta-analysis of prospective cohort studies. Medicine (Baltimore). 2016;95(33):e4229. doi: 10.1097/MD.0000000000004229. PubMed PMID: 27537552; PubMed Central PMCID: PMCPMC5370779.

16. O'Connor LE, Kim JE, Campbell WW. Total red meat intake of >/=0.5 servings/d does not negatively influence cardiovascular disease risk factors: a systemically searched meta-analysis of randomized controlled trials. Am J Clin Nutr. 2017;105(1):57-69. doi: 10.3945/ajcn.116.142521. PubMed PMID: 27881394; PubMed Central PMCID: PMCPMC5183733.

17. Zong G, Gao A, Hu FB, Sun Q. Whole Grain Intake and Mortality From All Causes, Cardiovascular Disease, and Cancer: A Meta-Analysis of Prospective Cohort Studies. Circulation. 2016;133(24):2370-80. doi: 10.1161/CIRCULATIONAHA.115.021101. PubMed PMID: 27297341; PubMed Central PMCID: PMCPMC4910651.

18. Qin LQ, Xu JY, Han SF, Zhang ZL, Zhao YY, Szeto IM. Dairy consumption and risk of cardiovascular disease: an updated meta-analysis of prospective cohort studies. Asia Pac J Clin Nutr. 2015;24(1):90-100. doi: 10.6133/apjcn.2015.24.1.09. PubMed PMID: 25740747.

19. Wang X, Lin X, Ouyang YY, Liu J, Zhao G, Pan A, et al. Red and processed meat consumption and mortality: dose-response meta-analysis of prospective cohort studies. Public Health Nutr. 2016;19(5):893-905. doi: 10.1017/S1368980015002062. PubMed PMID: 26143683.

20. Xi B, Huang Y, Reilly KH, Li S, Zheng R, Barrio-Lopez MT, et al. Sugar-sweetened beverages and risk of hypertension and CVD: a dose-response meta-analysis. Br J Nutr. 2015;113(5):709-17. doi: 10.1017/S0007114514004383. PubMed PMID: 25735740.

21. Zhan J, Liu YJ, Cai LB, Xu FR, Xie T, He QQ. Fruit and vegetable consumption and risk of cardiovascular disease: A meta-analysis of prospective cohort studies. Crit Rev Food Sci Nutr. 2017;57(8):1650-63. doi: 10.1080/10408398.2015.1008980. PubMed PMID: 26114864.

22. Abete I, Romaguera D, Vieira AR, Lopez de Munain A, Norat T. Association between total, processed, red and white meat consumption and all-cause, CVD and IHD mortality: a meta-analysis of cohort studies. Br J Nutr. 2014;112(5):762-75. doi: 10.1017/S000711451400124X. PubMed PMID: 24932617.

23. Afshin A, Micha R, Khatibzadeh S, Mozaffarian D. Consumption of nuts and legumes and risk of incident ischemic heart disease, stroke, and diabetes: a systematic review and meta-analysis. Am J Clin Nutr. 2014;100(1):278-88. doi: 10.3945/ajcn.113.076901. PubMed PMID: 24898241; PubMed Central PMCID: PMCPMC4144102.

24. Greenwood DC, Threapleton DE, Evans CE, Cleghorn CL, Nykjaer C, Woodhead C, et al. Association between sugar-sweetened and artificially sweetened soft drinks and type 2 diabetes: systematic review and dose-response meta-analysis of prospective studies. Br J Nutr. 2014;112(5):725-34. doi: 10.1017/S0007114514001329. PubMed PMID: 24932880.

25. Leung Yinko SS, Stark KD, Thanassoulis G, Pilote L. Fish consumption and acute coronary syndrome: a meta-analysis. Am J Med. 2014;127(9):848-57 e2. doi: 10.1016/j.amjmed.2014.04.016. PubMed PMID: 24802020.

26. Li YH, Zhou CH, Pei HJ, Zhou XL, Li LH, Wu YJ, et al. Fish consumption and incidence of heart failure: a meta-analysis of prospective cohort studies. Chin Med J (Engl). 2013;126(5):942-8. PubMed PMID: 23489806.

27. World Health Organization. Global strategy on diet, physical activity and health. 2004.

28. Miller V, Mente A, Dehghan M, Rangarajan S, Zhang X, Swaminathan S, et al. Fruit, vegetable, and legume intake, and cardiovascular disease and deaths in 18 countries (PURE): a prospective cohort study. Lancet. 2017;390(10107):2037-49. doi: 10.1016/S0140-6736(17)32253-5. PubMed PMID: 28864331.

29. Boeing H, Bechthold A, Bub A, Ellinger S, Haller D, Kroke A, et al. Critical review: vegetables and fruit in the prevention of chronic diseases. Eur J Nutr. 2012;51(6):637-63. doi: 10.1007/s00394-012-0380-y. PubMed PMID: 22684631; PubMed Central PMCID: PMCPMC3419346.

30. He FJ, Nowson CA, Lucas M, MacGregor GA. Increased consumption of fruit and vegetables is related to a reduced risk of coronary heart disease: meta-analysis of cohort studies. J Hum Hypertens. 2007;21(9):717-28. doi: 10.1038/sj.jhh.1002212. PubMed PMID: 17443205.

31. Messina V. Nutritional and health benefits of dried beans. Am J Clin Nutr. 2014;100 Suppl 1:437S-42S. doi: 10.3945/ajcn.113.071472. PubMed PMID: 24871476.

32. Ros E. Health benefits of nut consumption. Nutrients. 2010;2(7):652-82. doi: 10.3390/nu2070652. PubMed PMID: 22254047; PubMed Central PMCID: PMCPMC3257681.

33. Sabate J, Oda K, Ros E. Nut consumption and blood lipid levels: a pooled analysis of 25 intervention trials. Arch Intern Med. 2010;170(9):821-7. doi: 10.1001/archinternmed.2010.79. PubMed PMID: 20458092.

34. Eslamparast T, Sharafkhah M, Poustchi H, Hashemian M, Dawsey SM, Freedman ND, et al. Nut consumption and total and cause-specific mortality: results from the Golestan Cohort Study. Int J Epidemiol. 2017;46(1):75-85. doi: 10.1093/ije/dyv365. PubMed PMID: 26946539.

35. Guasch-Ferre M, Bullo M, Martinez-Gonzalez MA, Ros E, Corella D, Estruch R, et al. Frequency of nut consumption and mortality risk in the PREDIMED nutrition intervention trial. BMC Med. 2013;11:164. doi: doi:10.1186/1741-7015-11-164. PubMed PMID: 23866098; PubMed Central PMCID: PMCPMC3738153.

36. Yagi S, Fukuda D, Aihara KI, Akaike M, Shimabukuro M, Sata M. n-3 Polyunsaturated Fatty Acids: Promising Nutrients for Preventing Cardiovascular Disease. J Atheroscler Thromb. 2017;24(10):999-1010. doi: 10.5551/jat.RV17013. PubMed PMID: 28835582; PubMed Central PMCID: PMCPMC5656772.

37. Watanabe Y, Tatsuno I. Omega-3 polyunsaturated fatty acids for cardiovascular diseases: present, past and future. Expert Rev Clin Pharmacol. 2017;10(8):865-73. doi: 10.1080/17512433.2017.1333902. PubMed PMID: 28531360.

38. Bowen KJ, Harris WS, Kris-Etherton PM. Omega-3 Fatty Acids and Cardiovascular Disease: Are There Benefits? Curr Treat Options Cardiovasc Med. 2016;18(11):69. doi: 10.1007/s11936-016-0487-1. PubMed PMID: 27747477; PubMed Central PMCID: PMCPMC5067287.

39. Maehre HK, Jensen IJ, Elvevoll EO, Eilertsen KE. omega-3 Fatty Acids and Cardiovascular Diseases: Effects, Mechanisms and Dietary Relevance. Int J Mol Sci. 2015;16(9):22636-61. doi: 10.3390/ijms160922636. PubMed PMID: 26393581; PubMed Central PMCID: PMCPMC4613328.

40. Kris-Etherton PM, Harris WS, Appel LJ, Nutrition C. Fish consumption, fish oil, omega-3 fatty acids, and cardiovascular disease. Arterioscler Thromb Vasc Biol. 2003;23(2):e20-30. PubMed PMID: 12588785.

41. Elvevoll EO, Eilertsen KE, Brox J, Dragnes BT, Falkenberg P, Olsen JO, et al. Seafood diets: hypolipidemic and antiatherogenic effects of taurine and n-3 fatty acids. Atherosclerosis. 2008;200(2):396-402. doi: 10.1016/j.atherosclerosis.2007.12.021. PubMed PMID: 18242615.

42. Zheng J, Huang T, Yu Y, Hu X, Yang B, Li D. Fish consumption and CHD mortality: an updated meta-analysis of seventeen cohort studies. Public Health Nutr. 2012;15(4):725-37. doi: 10.1017/S1368980011002254. PubMed PMID: 21914258.

43. Lovegrove JA, Hobbs DA. New perspectives on dairy and cardiovascular health. Proc Nutr Soc. 2016;75(3):247-58. doi: 10.1017/S002966511600001X. PubMed PMID: 26907978.

44. Drouin-Chartier JP, Brassard D, Tessier-Grenier M, Cote JA, Labonte ME, Desroches S, et al. Systematic Review of the Association between Dairy Product Consumption and Risk of Cardiovascular-Related Clinical Outcomes. Adv Nutr. 2016;7(6):1026-40. doi: 10.3945/an.115.011403. PubMed PMID: 28140321; PubMed Central PMCID: PMCPMC5105032.

45. Dumas AA, Lapointe A, Dugrenier M, Provencher V, Lamarche B, Desroches S. A systematic review of the effect of yogurt consumption on chronic diseases risk markers in adults. Eur J Nutr. 2017;56(4):1375-92. doi: 10.1007/s00394-016-1341-7. PubMed PMID: 27807623.

46. Wu L, Sun D. Consumption of Yogurt and the Incident Risk of Cardiovascular Disease: A Meta-Analysis of Nine Cohort Studies. Nutrients. 2017;9(3). doi: 10.3390/nu9030315. PubMed PMID: 28327514; PubMed Central PMCID: PMCPMC5372978.

47. Micha R, Wallace SK, Mozaffarian D. Red and processed meat consumption and risk of incident coronary heart disease, stroke, and diabetes mellitus: a systematic review and meta-analysis. Circulation. 2010;121(21):2271-83. doi: 10.1161/CIRCULATIONAHA.109.924977. PubMed PMID: 20479151; PubMed Central PMCID: PMCPMC2885952.

48. Encuesta de Seguimiento al Consumo de Alimentos (ESCA). Caracas: Venezuela. Instituto Nacional de Estadística; 2014 2014//.

49. Singh GM, Micha R, Khatibzadeh S, Lim S, Ezzati M, Mozaffarian D, et al. Estimated Global, Regional, and National Disease Burdens Related to Sugar-Sweetened Beverage Consumption in 2010. Circulation. 2015;132(8):639-66. doi: 10.1161/CIRCULATIONAHA.114.010636. PubMed PMID: 26124185; PubMed Central PMCID: PMCPMC4550496.

50. Keller A, Heitmann BL, Olsen N. Sugar-sweetened beverages, vascular risk factors and events: a systematic literature review. Public Health Nutr. 2015;18(7):1145-54. doi: 10.1017/S1368980014002122. PubMed PMID: 25321082.
